# Geometrical Patterning and Constituent Cell Heterogeneity Facilitate Electrical Conduction Disturbances in a Human Induced Pluripotent Stem Cell-Based Platform: An *In vitro* Disease Model of Atrial Arrhythmias

**DOI:** 10.3389/fphys.2019.00818

**Published:** 2019-06-27

**Authors:** Hiroyuki Nakanishi, Jong-Kook Lee, Keiko Miwa, Kiyoshi Masuyama, Hideki Yasutake, Jun Li, Satoki Tomoyama, Yayoi Honda, Jiro Deguchi, Shinji Tsujimoto, Kyoko Hidaka, Shigeru Miyagawa, Yoshiki Sawa, Issei Komuro, Yasushi Sakata

**Affiliations:** ^1^Department of Cardiovascular Medicine, Graduate School of Medicine, Osaka University, Suita, Japan; ^2^Department of Advanced Cardiovascular Regenerative Medicine, Graduate School of Medicine, Osaka University, Suita, Japan; ^3^Department of Mechanical Engineering, Kyushu University, Fukuoka, Japan; ^4^Preclinical Research Unit, Sumitomo Dainippon Pharma Co., Ltd., Osaka, Japan; ^5^Regenerative & Cellular Medicine Office, Sumitomo Dainippon Pharma Co., Ltd., Osaka, Japan; ^6^Center for Fundamental Education, The University of Kitakyushu, Kitakyushu, Japan; ^7^Department of Cardiovascular Surgery, Graduate School of Medicine, Osaka University, Suita, Japan; ^8^Department of Cardiovascular Medicine, Graduate School of Medicine, The University of Tokyo, Tokyo, Japan

**Keywords:** human induced pluripotent stem cells, atrial-like cardiomyocytes, human atrial fibroblasts, geometrical patterning, constituent cell heterogeneity, electrical conduction, impaired conduction, source-to-sink mismatch

## Abstract

Ectopic foci from pulmonary veins (PVs) comprise the main trigger associated with the initiation of atrial fibrillation (AF). An abrupt anatomical narrow-to-wide transition, modeled as *in vitro* geometrical patterning with similar configuration in the present study, is located at the junction of PVs and the left atrium (LA). Complex cellular composition, i.e., constituent cell heterogeneity, is also observed in PVs and the PVs-LA junction. High frequency triggers accompanied with anatomical irregularity and constituent cell heterogeneity provoke impaired conduction, a prerequisite for AF genesis. However, few experiments investigating the effects of these factors on electrophysiological properties using human-based cardiomyocytes (CMs) with atrial properties have been reported. The aim of the current study was to estimate whether geometrical patterning and constituent cell heterogeneity under high frequency stimuli undergo conduction disturbance utilizing an *in vitro* two-dimensional (2D) monolayer preparation consisting of atrial-like CMs derived from human induced pluripotent stem cells (hiPSCs) and atrial fibroblasts (Fbs). We induced hiPSCs into atrial-like CMs using a directed cardiac differentiation protocol with the addition of all-*trans* retinoic acid (ATRA). The atrial-like hiPSC-derived CMs (hiPSC-CMs) and atrial Fbs were transferred in defined ratios (CMs/Fbs: 100%/0% or 70%/30%) on manually fabricated plates with or without geometrical patterning imitating the PVs-LA junction. High frequency field stimulation emulating repetitive ectopic foci originated in PVs were delivered, and the electrical propagation was assessed by optical mapping. We generated high purity CMs with or without the ATRA application. ATRA-treated hiPSC-CMs exhibited significantly higher atrial-specific properties by immunofluorescence staining, gene expression patterns, and optical action potential parameters than those of ATRA-untreated hiPSC-CMs. Electrical stimuli at a higher frequency preferentially induced impaired electrical conduction on atrial-like hiPSC-CMs monolayer preparations with an abrupt geometrical transition than on those with uniform geometry. Additionally, the application of human atrial Fbs to the geometrically patterned atrial-like hiPSC-CMs tended to further deteriorate the integrity of electrical conduction compared with those using the atrial-like hiPSC-CM alone preparations. Thus, geometrical narrow-to-wide patterning under high frequency stimuli preferentially jeopardized electrical conduction within *in vitro* atrial-like hiPSC-CM monolayers. Constituent cell heterogeneity represented by atrial Fbs also contributed to the further deterioration of conduction stability.

## Introduction

Atrial fibrillation (AF) comprises one of the most major arrhythmia worldwide, eliciting a critical impact on global health owing to the severe associated cardiovascular complications such as heart failure and thromboembolism, along with the increase in prevalence and incidence rate concomitant with the aging population (Miyasaka et al., 2006; Chugh et al., 2014; Rahman et al., 2014). It has been demonstrated that ectopic foci from pulmonary veins (PVs) constitute the main trigger associated with the initiation of AF (Haissaguerre et al., 1998). PVs exhibit relatively shorter effective refractory period (ERP) and slower conduction, providing a substrate for AF (Kumagai et al., 2004). The complicated electrophysiological properties in PVs result primarily from their anatomical complexity including the orientation and distribution of sleeve muscle fibers extending from the left atrium (LA) along with the composition of several types of local cells in the PV (Ho et al., 2001; Hocini et al., 2002; Hamabe et al., 2003). Microreentry is likely to be formed by impaired conduction and wavebreak due to the interaction of a wavefront with a barrier such as physical obstacle or electrical inhomogeneity (Waks and Josephson, 2014). Source-to-sink mismatch is shown to provoke conduction disturbance, and is more likely to occur in regions with an abrupt structural expansion (Rohr and Kucera, 1997; Rohr et al., 1997). It is supposed that intra-PVs and PVs-LA interface are more subject to such a geometrical effect in atrial chamber including PVs. PVs display complicated anatomical and electrophysiological properties as mentioned above, resulting in conduction disturbance and wavebreak based upon tissue discontinuities, followed by the initiation of AF within PVs. Actually, in radiofrequency catheter ablation of AF when it first came out, PV isolation was performed at each PV ostium, so-called “segmental ostial PV isolation,” and this procedure showed positive results to certain extent (Haissaguerre et al., 2000). On the other hand, sleeve muscle fibers exhibit an abrupt change in the direction and thickness at PVs-LA junction in sheep (Klos et al., 2008) and human hearts (Markides et al., 2003); moreover, it has been reported that PV impulses underwent conduction delay and wavebreak at the SPB of the PLA, as determined using three-dimensional (3D) mapping in patients with AF (Rha et al., 2008). This arrhythmogenicity at PVs-LA boundary is also supported by the evidence that circumferential PV isolation including PV antrum as isolation area was more effective in freedom from recurrent AF than segmental ostial PV isolation (Oral et al., 2003), and extensive encircling PV isolation including PV antrum and a part of PLA has recently become a dominant procedure of PV isolation (Ouyang et al., 2004). Therefore, it is considered that focal repetitive electrical activities at high frequency originating in PVs encounter anatomical irregularity (e.g., the abrupt structural changes causing source-to-sink mismatch) and local constituent cell heterogeneity in PVs and/or PVs-LA interface, subsequently undergoing impaired conduction and wavebreak, eventually resulting in the genesis of AF.

Rhythm control therapy for AF consists mainly of pharmacological intervention and catheter ablation procedures. Existing antiarrhythmic drugs for AF, however, are insufficient because of their limited effect on AF termination and the potential risk of unfavorable cardiac adverse events including bradycardias and ventricular arrhythmias. Thus, the construction of a pathophysiological model of AF and the establishment of a method for drug discovery exhibiting safety and efficacy as determined using human cell sources are of substantial importance.

Human induced pluripotent stem cells (hiPSCs) comprise a promising human-based cell source as a consequence of their pluripotency (Takahashi et al., 2007; Yu et al., 2007). A variety of cardiac differentiation protocols from hiPSCs have been reported, leading to the availability of hiPSC-derived cardiomyocytes (hiPSC-CMs) as an potent tool for cardiac regenerative therapy, development of disease-specific models, and drug screening systems (Burridge et al., 2012; Mummery et al., 2012). In turn, retinoic acid (RA), an endogenous retinol-derived morphogen that has critical roles during development (Marletaz et al., 2006; Cunningham and Duester, 2015), exerts specific effects on cardiogenesis inducing posteriorization of the embryonic heart, denoted by an enlarged atrium and reduced ventricle, when administered in excess, whereas deficient RA results in anteriorization, a smaller atrium and oversized ventricle (Xavier-Neto et al., 2001). Thus, exogenous RA has been demonstrated in several studies as being useful to induce CMs with atrial-like properties from various types of pluripotent stem cells, such as mouse embryonic stem cells (Hidaka et al., 2003), human embryonic stem cells (hESCs) (Zhang et al., 2011; Leyton-Mange et al., 2014; Devalla et al., 2015; Laksman et al., 2017; Lee et al., 2017), and hiPSCs (Lee et al., 2017; Cyganek et al., 2018; Lemme et al., 2018).

Previous studies have demonstrated that geometrical narrow-to-wide patterning promoted electrical conduction disturbance in an *in vitro* neonatal rat cardiomyocyte monolayer as a result of source-to-sink mismatch (Rohr and Kucera, 1997; Rohr et al., 1997; Kondratyev et al., 2007; Auerbach et al., 2011). On the other hand, the electrophysiological and pharmacological properties of atrial-like CMs derived from hESCs or hiPSCs have recently been elucidated (Leyton-Mange et al., 2014; Laksman et al., 2017; Lee et al., 2017). Therefore, it is important to develop a platform of human atrial arrhythmias using human-based atrial CMs with geometrical characteristics of PVs or PVs-LA junction. In addition, constituent cell heterogeneity represented by non-CMs including fibroblasts (Fbs) may also deteriorate the stability of electrical conduction. However, to our knowledge, little is known regarding the effects of geometrical patterning and constituent cell heterogeneity on electrical conduction in atrial-like hESC/hiPSC-CM preparations. In the current study, we focused on an abrupt change in *in vivo* 3D sleeve muscle thickness at PVs-LA interface, simplified it into a precipitous alteration in *in vitro* 2D strand width for the geometrical discontinuity. Moreover, we utilized human atrial Fbs as non-CMs for *in vitro* constituent cell heterogeneity. The purpose of the present study was therefore to ascertain whether geometrical patterning and constituent cell heterogeneity under electrical stimuli at high frequency provoked impaired electrical conduction, a prerequisite for the initiation of AF, in an *in vitro* 2D monolayer consisting of atrial-like hiPSC-CMs and human atrial Fbs.

## Results

### Cardiac Differentiation From hiPSCs and the Purity of hiPSC-Derived CMs

**FIGURE 1:**
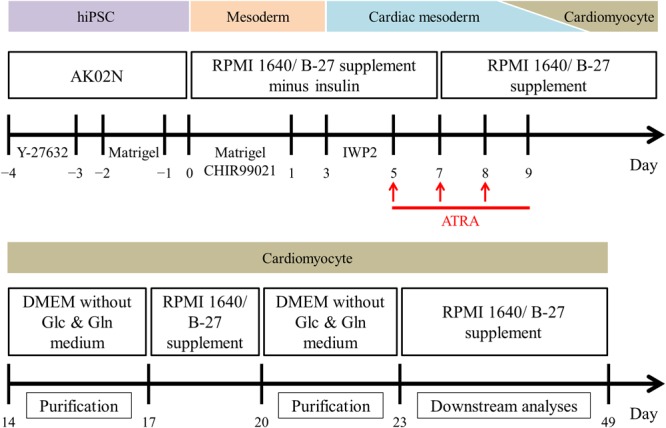
Overview of the monolayer-based directed cardiac differentiation protocol in the present study.

**FIGURE 2:**
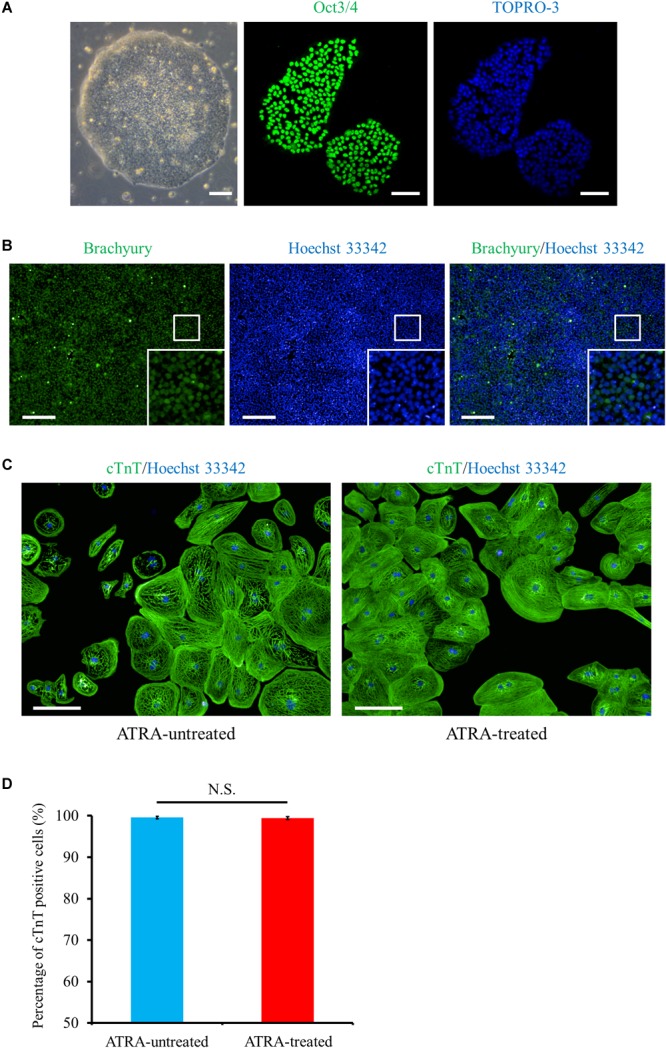
Cardiac differentiation through the mesoderm state from hiPSCs. **(A)** Phase contrast and immunofluorescence images of hiPSCs stained with a pluripotent marker, Oct3/4 (green). Scale bar, 50 μm (phase contrast), 100 μm (immunofluorescence). **(B)** Mesoderm marker (Brachyury, green) staining at day 1 post cardiac induction, i.e., just after 24 h exposure to CHIR99021. The bottom inset: high-magnification view of the region surrounded by the white square. Scale bar, 200 μm. **(C)** Immunofluorescence image of cTnT (green) under ATRA-untreated (control) (left panel) and ATRA-treated (right panel) conditions. Scale bar, 100 μm. **(D)** Quantitative analysis of the purity of CMs differentiated in the present protocol. The proportion of cTnT positive cells was 99.6 ± 0.3% (ATRA-untreated: control), or 99.5 ± 0.3% (ATRA-treated), respectively (*P* = N.S.). Error bars represent SD of the mean from the values of independent experiments (*n* = 6, each). N.S., not significant.

Using the method as shown in Figure 1, we differentiated the hiPSCs in a pluripotent state through the mesoderm stage into CMs (Figure 2A–C and Supplementary Figure 1). Quantitative immunofluorescence analysis using a high-content imaging system demonstrated that the protocol in the current study generated a high purity of CMs, positive for cardiac troponin T (cTnT), with no relation to the application of ATRA (cTnT positive cells; 99.6 ± 0.3 (ATRA-untreated) vs. 99.5 ± 0.3% (ATRA-treated), *n* = 6 (each), *P* = N.S., Figure 2D). Non-CMs were rarely observed in either ATRA-untreated or ATRA-treated groups, as indicated by the paucity of Fb or mesenchymal cell markers (Supplementary Figures 2A,B).

### Atrial Specific Properties in ATRA-Treated hiPSC-CMs

For atrial-specific commitment of hiPSC-CMs, we applied ATRA to the cardiac differentiation procedure during day 5 to 9. To verify the atrial specific properties in ATRA-treated hiPSC-CMs compared with those of ATRA-untreated hiPSC-CMs (control hiPSC-CMs), immunofluorescence staining, gene expression pattern analysis, and OAP morphology assessment using a voltage-sensitive dye were performed.

**FIGURE 3:**
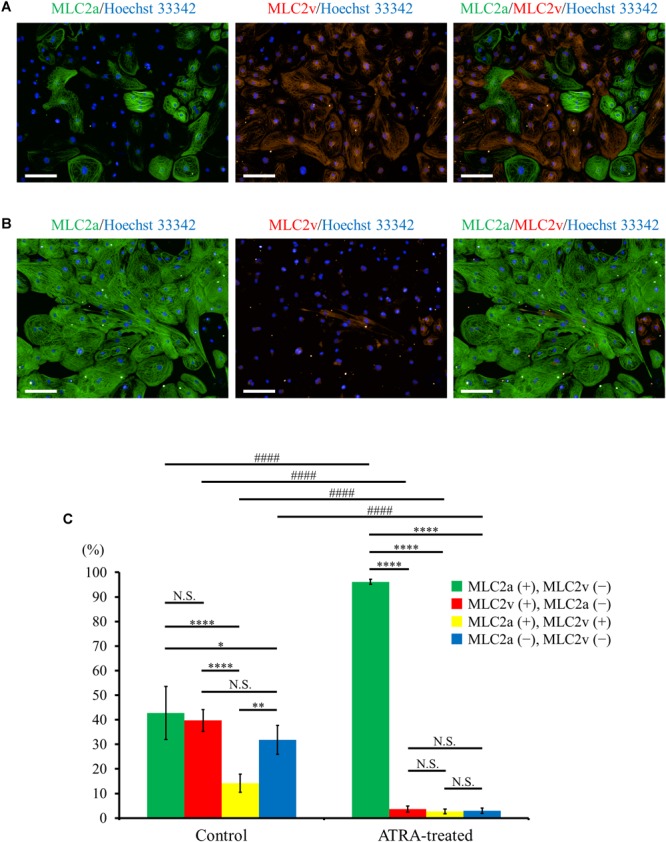
**(A,B)** Distribution of MLC2a (green) and MLC2v (red) expression in hiPSC-CMs under ATRA-untreated (control) **(A)** and ATRA-treated condition **(B)**. Scale bar, 100 μm. **(C)** Percentage of MLC2a and MLC2v positive expression in the two groups. Error bars represent SD of the mean from the values of independent experiments (*n* = 6, each). Different markers of each intragroup were analyzed using one-way ANOVA followed by Tukey’s *post hoc* test, and the same types of markers between the two groups were analyzed using unpaired *t*-test. N.S., not significant. ^∗^*P* < 0.05, ^∗∗^*P* < 0.01, ^∗∗∗∗^*P* < 0.0001 for each intragroup, respectively. ####*P* < 0.001 for control hiPSC-CMs vs. ATRA-treated CMs.

Immunofluorescence co-staining with myosin light chain (MLC) 2a and MLC2v demonstrated that MLC2a positive cells and MLC2v positive cells coexisted approximately to the same degree in control hiPSC-CMs (Figure 3A), whereas ATRA-treated CMs consisted almost entirely of MLC2a positive cells (Figure 3B). Quantitative immunofluorescence analysis using a high content imaging system revealed that MLC2a positive cells accounted for the most substantial fraction in ATRA-treated CMs (96.1 ± 1.0%, *P* < 0.0001, Figure 3C), although the expression levels of MLC2a and MLC2v in control hiPSC-CMs did not significantly differ (42.8 ± 10.8% vs. 39.7 ± 4.4%, *P* = N.S., Figure 3C). In addition, ATRA-treated CMs exhibited a higher proportion of MLC2a positive cells compared with that of control hiPSC-CMs (96.1 ± 1.0% vs. 42.8 ± 10.8%, *P* < 0.0001, Figure 3C), albeit a lower proportion of MLC2v positive cells than that in the control group (3.6 ± 1.2% vs. 39.7 ± 4.4%, *P* < 0.0001, Figure 3C).

**FIGURE 4:**
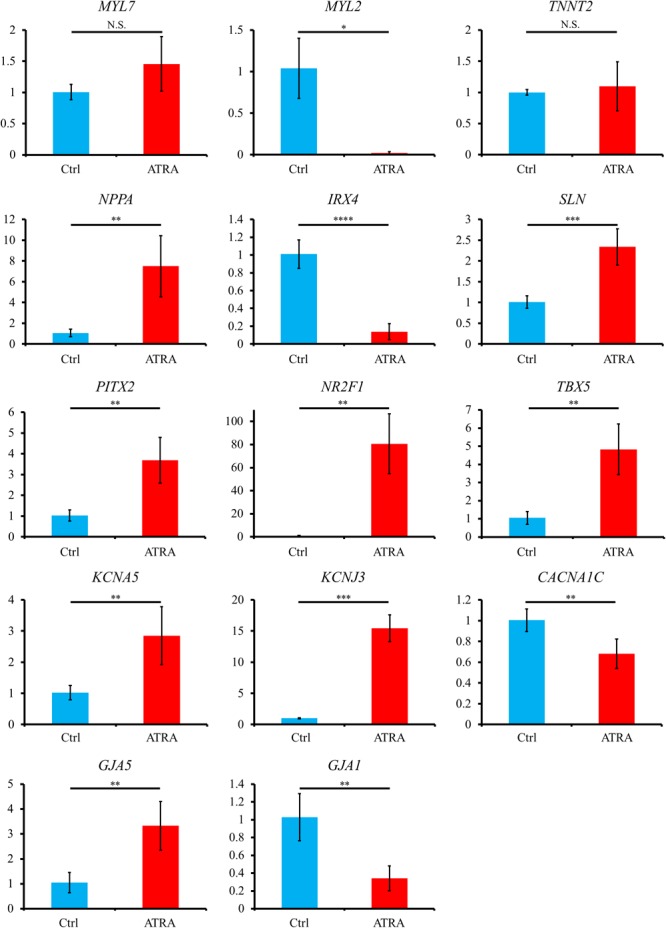
Gene expression analyses via qRT-PCR between control and ATRA-treated hiPSC-CMs. Data are presented as the means ± SD. *PITX2, NR2F1, TBX5*: *n* = 5 (ctrl) vs. 5 (ATRA); other genes: *n* = 4 (ctrl) vs. 5 (ATRA). N.S., not significant. ^∗^*P* < 0.05, ^∗∗^*P* < 0.01, ^∗∗∗^*P* < 0.001, ^∗∗∗∗^*P* < 0.0001 for control vs. ATRA-treated CMs. Ctrl, control hiPSC-CMs (ATRA-untreated hiPSC-CMs); ATRA, ATRA-treated hiPSC-CMs. Vertical axis denotes relative gene expression.

Quantitative reverse transcription polymerase chain reaction (qRT-PCR) exhibited that atrial specific genes such as *NR2F1, PITX2, TBX5, NPPA, SLN, KCNA5, KCNJ3*, and *GJA5* were significantly upregulated in ATRA-treated hiPSC-CMs compared with those in control hiPSC-CMs, although no significant difference between the two groups was observed for an atrial marker, *MYL7*, despite a slight tendency toward higher value in ATRA-treated hiPSC-CMs (*P* = 0.0885) (Figure 4). In contrast, ventricular specific genes such as *MYL2, IRX4*, and *GJA1* were significantly downregulated in ATRA-treated hiPSC-CMs compared to those in control hiPSC-CMs (Figure 4). The expression of *TNNT2* did not significantly differ between the two groups, suggesting that a similar purity of CMs was also observed at the level of gene expression (Figure 4). A significant downregulation of *CACNA1C* coding Ca_v_1.2, a component of the plateau phase of cardiac action potential, was observed in ATRA-treated hiPSC-CMs compared to that of control hiPSC-CMs (Figure 4). In comparison, ATRA-treated hiPSC-CMs expressed significantly higher levels of nodal-specific genes, *SHOX2* and *HCN4* (Supplementary Figure 3). The expression levels of the other tested genes are shown in Supplementary Figure 3.

**FIGURE 5:**
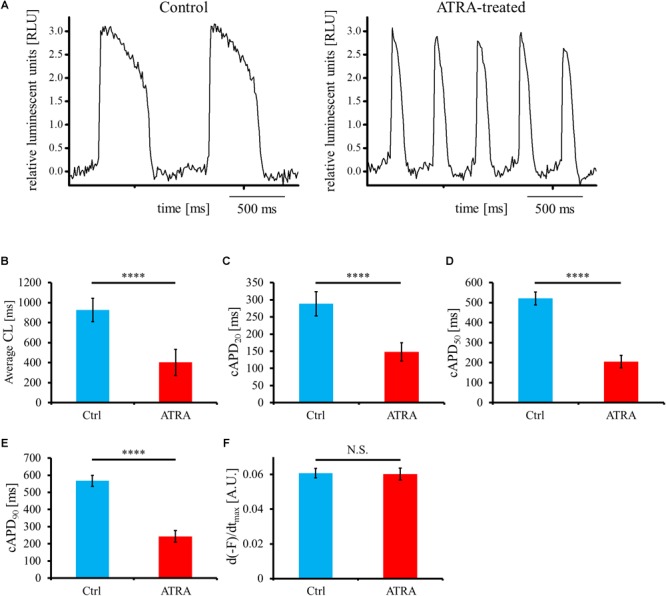
**(A)** Representative OAP morphology in control (left panel) and ATRA-treated hiPSC-CMs (right panel). Time scale bar, 500 ms. RLU, relative luminescent units. **(B–F)** Optical action potential (OAP) parameters including average CL **(B)**, cAPD_20_ **(C)**, cAPD_50_ **(D)**, cAPD_90_ **(E)**, and d(-F)/dt_max_ **(F)** using voltage-sensitive dye between control and ATRA-treated hiPSC-CMs. Data are presented as the means ± SD. *n* = 9 (ctrl) vs. 8 (ATRA). N.S., not significant. ^∗∗∗∗^*P* < 0.0001 for control hiPSC-CMs vs. ATRA-treated CMs. A.U., arbitrary unit; Ctrl, control hiPSC-CMs (ATRA-untreated hiPSC-CMs); ATRA, ATRA-treated hiPSC-CMs.

For the estimation of electrical phenotype, OAP parameters such as average cycle length (CL), corrected action potential duration (cAPD), and maximum upstroke velocity of OAP (d(-F)/dt_max_) were analyzed from the acquired OAP waveforms (Figure 5A). Average CL was significantly shorter in ATRA-treated hiPSC-CMs than that in control hiPSC-CMs (control vs. ATRA = 927.6 ± 116.4 vs. 402.5 ± 130.0 ms, *n* = 9 vs. 8, respectively, *P* < 0.0001, Figure 5B). ATRA-treated hiPSC-CMs exhibited significantly shorter cAPD_x_ (*x* = 20, 50, 90) compared with that of control hiPSC-CMs (control vs. ATRA = cAPD_20_: 288.4 ± 35.4 vs. 148.2 ± 26.9 ms, cAPD_50_: 521.1 ± 32.6 vs. 205.4 ± 31.5 ms, cAPD_90_: 567.1 ± 31.7 vs. 243.7 ± 33.5 ms, *n* = 9 vs. 8, respectively, *P* < 0.0001 for all, Figure 5C–E). There was no significant difference in d(-F)/dt_max_ between the two groups (control vs. ATRA = 0.061 ± 0.003 vs. 0.060 ± 0.004 A.U., *n* = 9 vs. 8, respectively, *P* = N.S., Figure 5F).

Together, these findings indicated that ATRA-treated hiPSC-CMs obtained more atrial-like properties compared with those of control hiPSC-CMs.

### Impaired Electrical Conduction in the Geometrical Patterning Model

**FIGURE 6:**
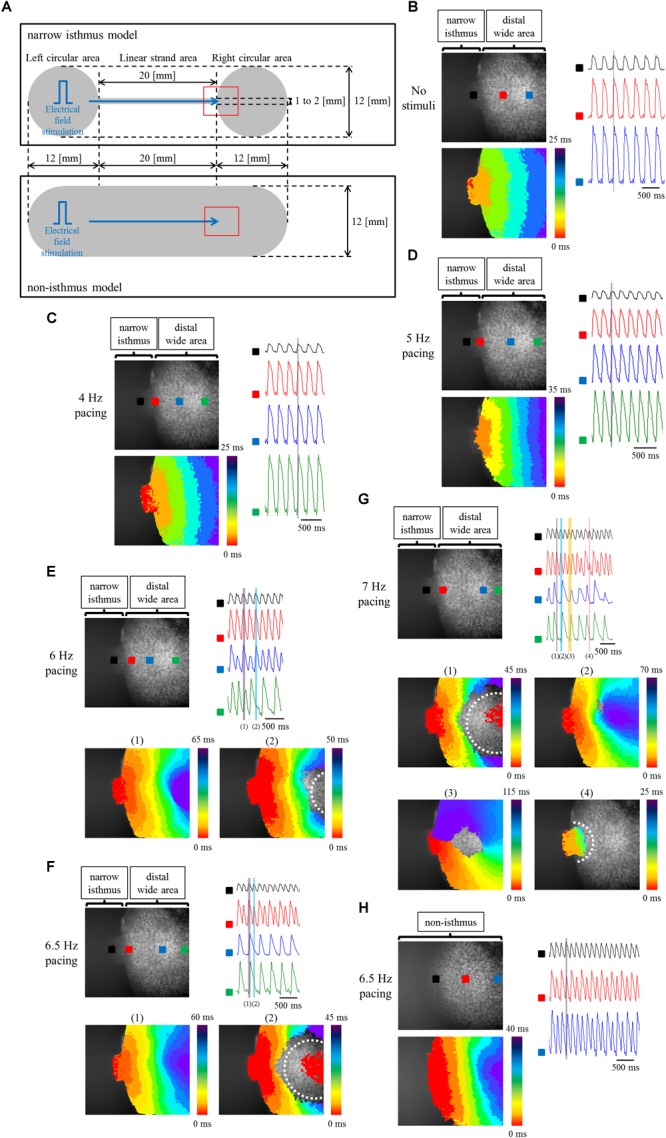
**(A)** Frame design of the fabricated plates in the narrow isthmus model (upper panel) and non-isthmus model (lower panel). The recording site of optical mapping is illustrated by the hollow red square. Blue arrow indicates the direction of electrical conduction generated by electrical field stimuli. **(B–G)** Optical mapping of a narrow isthmus model containing atrial-like hiPSC-CMs alone under spontaneous beating with no electrical stimuli **(B)**, constant pacing at 4 Hz **(C)**, 5 Hz **(D)**, 6 Hz **(E)**, 6.5 Hz **(F)**, and 7 Hz **(G)**. 1 to 1 conduction **(C,D)**, conduction delay **[E-(1)]** and intermittent regional block (crab claw-like conduction) **[E-(2)]**, 2:1 conduction block **[F-(1)**: conduction delay, **F-(2)**: regional block (crab claw-like conduction)**]**, various types of conduction disturbance (**G-(1)**: regional block (crab claw-like conduction), **G-(2)**: conduction delay, **G-(3)**: unidirectional block, **G-(4)**: omnidirectional block) were observed. **(B-G upper left panel)** The recorded field in the preparation. OAP waveforms were acquired at each ROI as indicated by a solid square. **(B–D lower left panel, E,F lower panel, G middle and lower panel)** Activation map on the recorded field during the time range denoted by the light purple, light blue, light orange, or light pink rectangle in the right (upper) panel. White dotted line indicates the estimated conduction block line. Color bar denotes total activation time (ms). **(B–D right panel, E–G right upper panel)** Representative OAP waveforms acquired at each ROI as indicated by a solid square in the upper left panel. Light purple, light blue, light orange, or light pink rectangle designates the time range during which the activation map was developed. Time scale bar, 500 ms. **(H)** Optical mapping of a non-isthmus model containing atrial-like hiPSC-CMs alone under constant pacing at 6.5 Hz. 1 to 1 conduction was maintained at 6.5 Hz. (Upper left panel) The recorded field in the preparation. OAP waveforms were acquired at each ROI as indicated by a solid square. (Lower left panel) Activation map on the recorded field during the time range denoted by the light purple rectangle in the right panel. Color bar denotes total activation time (ms). (Right panel) Representative OAP waveforms acquired at each ROI as indicated by a solid square in the upper left panel. Light purple rectangle designates the time range during which the activation map was developed. Time scale bar, 500 ms. Please be careful not to misunderstand the indication of the activation mapping in **F-(2)** and **G-(1)**: the red-colored activation pattern at the center of the blocked zone does not mean competition with an emerging focus within the blocked zone, but is caused by the detection of the previous OAP intensity at the late phase of repolarization at the ROI. Please note that the light blue rectangle in the right upper panel of **F** and the light purple rectangle in the right upper panel of **G** are correspond to the timing of the late repolarization phase of the each previous OAP at the ROI.

To elucidate the effects of geometrical narrow-to-wide patterning on electrical conduction, atrial-like hiPSC-CMs were plated on the fabricated plate, which contained a narrow isthmus structure with an abrupt geometrical transition or non-isthmus structure with uniform geometry (Figure 6A). Initially, optical mapping was performed under spontaneous beating without any electrical field stimuli. As an example, spontaneous electrical activity in a narrow isthmus model was propagated from the extreme left of the narrow strand area within the field of view, suggesting that the earliest activation site during the spontaneous rhythm in the preparation originated from a zone further left outside the field (Figure 6B and Supplementary Movie 1). Next, electrical field stimuli were delivered into the pacing site. Under the pacing frequency at which 1 to 1 conduction within the field was sustained, electrical excitation generated from the pacing area was robustly propagated through the narrow isthmus into the right circular area (Figure 6C,D and Supplementary Movies 2, 3). When pacing rate was further increased, intermittent regional block (crab claw-like conduction) (Figure 6E and Supplementary Movie 4), 2:1 conduction block (Figure 6F and Supplementary Movie 5), or sequential types of various conduction disturbance (regional block (crab claw-like conduction), unidirectional block, or omnidirectional block) (Figure 6G and Supplementary Movie 6) along with conduction delay (Figure 6E–G) were observed at rather distal from narrow-to-wide interface in the narrow isthmus model, although 1 to 1 electrical conduction into the distal area was sustained even at 6.5 Hz constant pacing in the non-isthmus model (Figure 6H and Supplementary Movie 7). Conduction disturbance became more evident particularly at higher pacing frequency (Figure 6G and Supplementary Movie 6). In addition, electrical alternans in OAP amplitude and duration was detected typically at higher pacing condition (Figure 6F,H). According to the criteria of the success rate of electrical conduction into the distal region as described in Section “Optical Mapping,” the success rate was preferentially decreased in the narrow isthmus model compared with that in the non-isthmus model, and the tendency became prominent depending on the increase in pacing frequency (Table 1). Therefore, optical mapping revealed that electrical stimuli at a higher frequency delivered to the atrial-like hiPSC-CM monolayer preparation preferentially induced impaired electrical conduction on the narrow isthmus model with the abrupt geometrical transition patterning compared with that on the non-isthmus model without any geometrical change.

**Table 1:** Success rate of electrical conduction into the distal region at each pacing frequency between the narrow isthmus and non-isthmus model.

Pacing frequency (Hz)	5	5.5	6	6.5	7
Non-isthmus model (*n* = 3)	100	100	100	100	33.3
Narrow isthmus model (*n* = 4)	100	75	25	0^∗^	0

*All preparations consisted of atrial-like hiPSC-CMs alone. Data are indicated by percentage (%). The comparison of the success rate at each pacing frequency was analyzed using Fisher’s exact test. ^∗^*P* < 0.05 compared with the non-isthmus model.*

### Constituent Cell Heterogeneity With Atrial Fbs Further Deteriorates the Integrity of Electrical Conduction

**FIGURE 7:**
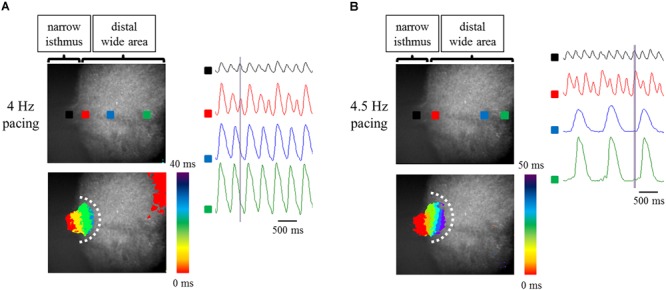
**(A,B)** Optical mapping of a narrow isthmus model containing 70% atrial-like hiPSC-CMs and 30% Fbs under constant pacing at 4 Hz **(A)** and 4.5 Hz **(B)**. Omnidirectional block was observed **(A,B)**. (Upper left panel) The recorded field in the preparation. OAP waveforms were acquired at each ROI as indicated by a solid square. (Lower left panel) Activation map on the recorded field during the time range denoted by the light purple rectangle in the right panel. White dotted line indicates the estimated conduction block line. Color bar denotes total activation time (ms). (Right panel) Representative OAP waveforms acquired at each ROI as indicated by a solid square in the upper left panel. Light purple rectangle designates the time range during which the activation map was developed. Time scale bar, 500 ms.

It has been reported that Fbs constitute one of the major components of non-CMs in the human heart (Bergmann et al., 2015). Thus, to examine the effects of constituent cell heterogeneity on electrical conduction, human atrial Fbs were employed. The Fbs, positive for vimentin and negative for cTnT (Supplementary Figure 4), were co-cultured with atrial-like hiPSC-CMs on the fabricated plate with a narrow isthmus. The preparations consisting of 70% atrial-like hiPSC-CMs and 30% Fbs resulted in conduction disturbance on the slightly forward region of the narrow-to-wide transition at lower pacing frequency relative to the atrial-like hiPSC-CM alone preparation (Figure 7A,B, Table 2 and Supplementary Movies 8, 9). Electrical alternans in OAP amplitude and duration was also observed (Figure 7B). As predicted, the preparation containing only Fbs exhibited quiescent electrical activity with or without electrical stimuli (Supplementary Figure 5 and Supplementary Movies 10, 11).

**Table 2:** Success rate of electrical conduction into the distal region at each pacing frequency between atrial-like hiPSC-CMs alone and atrial-like hiPSC-CMs/Fbs coculture preparations with geometrical patterning.

Pacing frequency (Hz)	4	4.5	5	5.5	6	6.5	7
CMs alone (*n* = 4)	NA	NA	100	75	25	0	0
CMs/Fbs coculture (*n* = 3)	66.7	66.7	0^∗^	0	0	0	0

*Coculture preparations consisted of 70% atrial-like hiPSC-CMs and 30% human atrial Fbs. Data are indicated by percentage (%). The comparison of the success rate at each pacing frequency was analyzed using Fisher’s exact test. ^∗^*P* < 0.05 compared with atrial-like hiPSC-CMs alone preparations. NA indicates the preparations to which electrical stimuli were unable to be delivered at the pacing frequency owing to the higher spontaneous beating rate than the pacing rate.*

These results denoted that the application of human atrial Fbs to the atrial-like hiPSC-CMs on the fabricated plate with geometrical narrow-to-wide patterning deteriorated the stability of electrical conduction in a milder pacing condition compared with that observed with the atrial-like hiPSC-CM alone preparation.

## Discussion

In the present study, we demonstrated that geometrical patterning with an abrupt narrow-to-wide transition mimicking the anatomical architecture of the human PVs-LA junction preferentially impaired electrical conduction in an *in vitro* atrial-like hiPSC-CM 2D monolayer preparation under electrical stimuli at higher frequency compared with that obtained in a uniform geometry model. In addition, constituent cell heterogeneity with non-CMs represented by human atrial Fbs further deteriorated the integrity of electrical conduction relative to that of the atrial-like hiPSC-CM alone preparation. In comparison, the majority of *in vitro* experiments featuring source-to-sink mismatch have been implemented in neonatal rat CMs (Rohr and Kucera, 1997; Rohr et al., 1997; Kondratyev et al., 2007; Auerbach et al., 2011). To our knowledge, few studies have investigated electrophysiological behavior, especially using an atrial-like hESC/hiPSC-CM preparation with geometrical patterning to elicit source-to-sink mismatch, although numerous reports exist regarding *in vitro* electrophysiological studies using hESC/hiPSC-CMs free from abrupt structural change, including those with an atrial-like phenotype.

According to the concept of source-to-sink mismatch, electrical conduction is more likely to be disrupted when the current source (i.e., the supply of upstream depolarizing cells) is relatively small as compared with current load (i.e., the demand of downstream depolarized cells) (Rohr and Kucera, 1997; Rohr et al., 1997; Kondratyev et al., 2007; Auerbach et al., 2011). *In vivo* (including in the human heart) PVs contain sleeve muscle fibers that extend from the LA, with these fibers showing complex anisotropy and discontinuity (Ho et al., 2001; Hocini et al., 2002; Hamabe et al., 2003). The peripheral ending of myocardial sleeves in human PVs consists of encapsulated small groups of CMs within fibrous tissues (Saito et al., 2000). Such anisotropy and discontinuity of fibers attenuates electrical coupling conductance and suppresses the electrotonic loading effects of the surrounding cells, followed by the stability of ectopic focal activity (Wilders et al., 2000). Thus, the sleeve muscle fibers in PVs are considered to play a role in the genesis of ectopic foci with repetitive high frequency. It is supposed that intra-PVs and PVs-LA interface is favored site of source-to-sink mismatch in atrial chamber including PVs, due to the complicated anatomical and electrophysiological properties in PVs, and the abrupt thickness and direction of sleeve myocardium at PVs-LA junction, respectively. Actually, repetitive electrical activities at high frequency originating in PVs undergo conduction disturbance and wavebreak at the PVs-LA interface as a consequence of source-to-sink mismatch, leading to AF initiation (Klos et al., 2008). The cell alignment and distribution pattern in PVs displays a highly individual variation (Ho et al., 2001), so we thought that it was difficult for us to construct an *in vitro* atrial arrhythmia model faithfully replicating intra-PVs arrhythmogenicity. Alternatively, we focused on an abrupt change in *in vivo* 3D sleeve muscle thickness at PVs-LA interface, simplified it into a precipitous alteration in *in vitro* 2D strand width. In the present study, geometrical transition was patterned in a manner with a narrow isthmus connected to a wide expansion area, imitating the condition of a small current source to large current load at PVs-LA boundary with an abrupt increase in thickness. High frequency electrical field stimuli, emulating high frequency PV triggers, underwent impaired conduction, including conduction delay or block through the abrupt geometrical expansion. Therefore, these findings suggested that source-to-sink mismatch also underlies the electrical conduction disturbance in the *in vitro* atrial-like hiPSC-CM preparation with geometrical patterning.

Constituent cell heterogeneity, represented by the composition of several types of cells, also contributes to the complicated electrophysiological properties in PVs (Ho et al., 2001; Hamabe et al., 2003). The adult mammalian heart consists of approximately 30% CMs and 70% non-CMs (Baudino et al., 2006), with cardiac Fbs being shown to constitute a major component of non-CMs in the human heart (Bergmann et al., 2015). Wavebreak, a critical factor of AF initiation, is likely to be generated when a rapid electrical wavefront collides with an anatomical or functional obstacle in an adequate condition (Vaquero et al., 2008). In addition, electrotonic coupling of CMs with non-excitable cells such as Fbs imposes on the CMs the slowing of the maximum upstroke velocity during phase 0 of the cardiac action potential and the shortening of action potential duration during phase 3, leading to a conduction delay and ERP shortening (Yue et al., 2011). Therefore, atrial Fbs are considered to have some influence on the arrhythmogenesis in atrium including PVs as electrotonic loads as well as mere physical barriers.

In the present study, we succeeded in the generation of high purity (>95%) hiPSC-CMs with or without ATRA treatment (Figure 2D). High purity of hiPSC-CMs is required to minimize the influences of non-CMs and to conduct proper electrophysiological experiments. As it has been well documented that CMs derived from human pluripotent stem cells contain various phenotypes including nodal-, atrial-, and ventricular-like (Blazeski et al., 2012), the induction of CMs with a defined fraction is mandatory for the development of a cardiac chamber-specific pathophysiological model. In our experiment, ATRA-treated hiPSC-CMs exhibited atrial-like properties in immunofluorescence staining (Figure 3A–C), gene expression pattern (Figure 4), and OAP morphology (Figure 5A–F) assays, with these atrial specific characteristics being highly consistent with those in previous reports (Zhang et al., 2011; Leyton-Mange et al., 2014; Devalla et al., 2015; Laksman et al., 2017; Lee et al., 2017).

There are several limitations in the current study. Firstly, we did not succeed in the induction of AF in our setting, although rapid burst pacing was delivered to induce fibrillatory activity, referring to the recent report in which AF was inducible on an atrial-like hESC-CM sheet without any geometrical patterning (Laksman et al., 2017). Impaired conduction such as conduction delay and unidirectional block as well as shortening of ERP is a prerequisite for the formation of reentry (Waks and Josephson, 2014). Augmentation of source-to-sink mismatch, which represents a much smaller current source relative to a far larger current load, theoretically aggravates the robustness of electrical conduction. Typical pattern of conduction disturbance based upon geometrical discontinuity has been shown to occur at or close to narrow-to-wide interface (Fast and Kleber, 1995; Rohr and Kucera, 1997; Auerbach et al., 2011), and displayed convex wavefront curvature during conduction disturbance (Rohr and Kucera, 1997; Auerbach et al., 2011). Certainly, narrow strand width in the current study was rather wider than that of the previous studies (Fast and Kleber, 1995; Rohr and Kucera, 1997), although it was technically difficult to engrave microgroove manually in the present study. We speculate that relatively wider width of narrow isthmus in our study attenuated source-to-sink mismatch effect, resulting in conduction disturbance at more distal area beyond narrow-to-wide interface and with concave wavefront curvature in atrial-like hiPSC-CM alone preparations. Actually, in the Rohr’s study, narrow strand width was defined as 10s of μm, and the conduction disturbance occurred at the boundary between narrow strand and wide expansion area (Rohr and Kucera, 1997). Fast VG reported that narrow strand width of approximately 15 μm enabled the induction of conduction disturbance at narrow-to-wide interface without high frequency pacing (Fast and Kleber, 1995). In addition, biphasic upstroke waveform of action potential reflecting narrow-to-wide geometrical effect as seen in the previous study (Fast and Kleber, 1995) was not observed in our experiment, although it was likely due to a rather low sampling rate as well as a mild source-to-sink mismatch effect in our study. However, as we showed in Table 1, non-isthmus model had a greater tolerance for conduction disturbance induced by high frequency pacing compared with narrow isthmus model. Therefore, it is suggested that pacing frequency-dependent conduction disturbances at the more distal area from narrow-to-wide interface and with concave wavefront on atrial-like hiPSC-CM alone preparations were evoked by complementary effect of a mild source-to-sink mismatch and local dispersion of refractory period under high frequency pacing. On the other hand, as shown in Figure 7A,B, coculture preparations with cardiac fibroblasts displayed convex wavefront curvature during conduction failure, and the conduction block occurred close to narrow-to-wide interface. We assume that fibroblasts acted as a barrier such as physical obstacle or electrical heterogeneity, resulting in the augmentation of narrow-to-wide geometrical effect. A finer microstructure model using photolithography technique may be useful for the analysis of pure source-to-sink mismatch effect also on *in vitro* atrial-like hiPSC-CMs in the future. Moreover, the simplified 2D model used in the present experiment lacked several factors present *in vivo* such as the cell direction and fiber thickness, the distribution of extracellular matrix, autonomic innervation, the electrophysiological discrepancy between PV and atrial cardiomyocytes, and cardiac overload, so it is unclear to what extent the narrow isthmus width and the ratio of narrow strand width to wide expansion one in the present study was appropriate for the arrhythmogenicity in PVs-LA boundary, although in the 3D reconstruction study in sheep atrial chamber including PVs, PV sleeve myofibers show thin-walled structure distal to the PVs-LA junction, followed by an abrupt increase in thickness at the PVs-LA interface (change from approximately 1 to 4 mm) (Zhao et al., 2012). Considering that an abrupt change in fiber direction and thickness at the PV-SPB transition constitutes a potent contributor to arrhythmogenesis (Klos et al., 2008), a more elaborate 3D model incorporating these properties is likely to facilitate the genesis of AF in the future. In addition, PV myocardium has been shown to have different electrophysiological properties from LA cardiomyocyte. Although the electrophysiological studies of isolated PV myocardium have been performed mainly on non-human mammal, PV cardiomyocytes displayed significantly shorter APD_50_ and APD_90_, significantly less negative resting membrane potential (RMP), significantly reduced dV/dt_max_ compared with those of LA cardiomyocytes (Ehrlich et al., 2003; Okamoto et al., 2012). Inward rectifier current (*I*_K1_) in PV cardiomyocytes were significantly smaller than that of LA cardiomyocytes, leading to the less negative RMP in PV myocardium (Ehrlich et al., 2003). Smaller L-type calcium current (*I*_Ca,L_) and greater delayed rectifier K^++^ current (*I*_Ks_ and *I*_Kr_) in PV cardiomyocytes contribute to the shorter APD in PV myocardium (Ehrlich et al., 2003). According to a clinical electrophysiological study on patients with paroxysmal AF, the longest conduction time from PV distal to PV-LA junction was 114 ± 46 ms, which means a considerable conduction delay in PVs (Kumagai et al., 2004). Lower expression of connexin 40 in PVs also resulted in delayed conduction in PVs (Verheule et al., 2002). These electrophysiological properties in PVs are thought to contribute to the arrhythmogenicity. It has been recently elucidated that PV myocardium derives from mesenchyme around the PV, and *Pitx2* and *Nkx2.5* play a key role in the development process (Mommersteeg et al., 2007; Christoffels et al., 2010). In addition, low expression of *PITX2* and *NKX2.5* induces PV myocardium into nodal-like cells, and functional insufficiency of *PITX2* is involved with an increased risk of AF (Franco et al., 2014). However, to our knowledge, the differentiation method dedicated to the induction into PV myocardium from hESCs or hiPSCs has not been developed, so it remains difficult to construct an *in vitro* arrhythmia model consisting of distinct atrial-like CMs and PV-like myocardium derived from hESCs or hiPSCs.

Secondly, a considerably high pacing frequency beyond physiological range was required to induce conduction disturbance in our setting. As shown in Figure 5B, the spontaneous beating cycle length of ATRA-treated hiPSC-CMs in the present study was 402.5 ms (in other words, approximately 150 bpm), which was higher than the normal heart rate in human. As elaborated below, an increase in the beating rate was considered to be mainly due to the existence of nodal-like population in our ATRA-treated hiPSC-CMs, and the increased automaticity was thought to contribute to the development of resistance to impaired conduction caused by pacing stimuli. However, electrical stimuli with markedly short cycle length are useful for the induction of conduction disturbance and/or wavebreak, and the adequate frequency depends on the cell inherent electrophysiological properties including spontaneous beating rate. Actually, coupling interval of premature beats originated in PVs has been shown to be far shorter compared with the normal spontaneous beating interval in human heart. In a clinical electrophysiological study on patients with paroxysmal AF, the ERP of the PVs and PVs-LA junction were 177 and 222 ms, respectively (Kumagai et al., 2004), allowing extrasystoles with a quite short coupling interval, followed by impaired conduction and/or wavebreak. Atrial-like hiPSC-CMs with almost the same characteristics as adult human atrial cardiomyocytes would facilitate electrophysiological experiments more relevant to human atrial arrhythmias. Moreover, electrical alternans was seen in OAP amplitude and duration, and this phenomenon tended to emerge with the increase in pacing rate, as typically observed in Figure 6F,H, 7B. It is recognized that cardiac alternans at the cellular level such as APD alternans and Ca^2+^ alternans can be caused by the interactive coupling deterioration between membrane voltage and intracellular calcium, and these alternans is likely to be seen particularly under constant high frequency pacing (Edwards and Blatter, 2014). Activation of L-type Ca^2+^ channel under high rate pacing stimuli can facilitate alternans via the steepening of APD restitution slope and the impaired regulation of intracellular Ca^2+^ handling. OAP alternans in our setting characterized by long OAP duration with high amplitude and short OAP duration with low amplitude was consistent with the previous report (de Diego et al., 2008). It is suggested in the present study that a long OAP duration shortened diastolic interval (DI), and the shortened DI could not provide sufficient time for the recovery of L-type Ca^2+^ channel, followed by the reduction in L-type Ca^2+^ channel availability. Consequently, the following beat was thought to exhibit short OAP duration. On the other hand, it is also suggested that the short OAP duration prolonged DI, and the prolonged DI enabled the sufficient recovery of L-type Ca^2+^ channel, resulting in the prolongation of OAP duration during the next beat. Although it is difficult to explain the mechanism responsible for the alternans in OAP amplitude, it is speculated that incomplete recovery of ion channels in a state of inactivation associated with action potential had an influence on the amplitude alternans. In addition, it has been reported in clinical setting that atrial alternans in action potentials is a risk factor of AF (Narayan et al., 2002). Therefore, pacing-induced OAP alternans in our study might reflect a proarrhythmic state for atrial arrhythmias.

Thirdly, it has been widely recognized that hESC/hiPSC-CMs exhibit a more immature phenotype compared with that of adult human CMs, affecting the electrophysiological properties (Feric and Radisic, 2016). The gene expression pattern of ion channels in hESC-CMs displayed fetal-like properties, represented by the lower *SCN5A, KCNJ2*, and the higher *HCN4*, resulting in slower upstroke velocity, less negative RMP, and automaticity (Sartiani et al., 2007; Laksman et al., 2017). According to a previous report using patch-clamp technique, APD_20_, APD_50_, APD_90_, and dV/dt_max_ in atrial-like hESC-CMs showed 20.8 ± 3.7 ms, 44 ± 10 ms, 145 ± 21 ms, 26.3 ± 2.7 V/s, respectively, while APD_20_, APD_50_, APD_90_, and dV/dt_max_ in ventricular-like hESC-CMs exhibited 82.0 ± 15.9 ms, 132 ± 21 ms, 181 ± 30 ms, 50.4 ± 10.8 V/s, respectively (Devalla et al., 2015). Conduction velocity in hiPSC-CM sheets was approximately 20 cm/s (Lee et al., 2012). On the other hand, APD_30_, APD_50_, APD_90_, and dV/dt_max_ in isolated adult human atrial cardiomyocytes were 10 ± 13 ms, 45 ± 79 ms, 383 ± 103 ms, and 172 ± 60 V/s, respectively (Dawodu et al., 1996), while APD_60_, APD_90_, and dV/dt_max_ in isolated adult human ventricular cardiomyocytes were respectively 270 to 365 ms, 330 to 439 ms, and 228 to 326 V/s with transmural differences observed (Drouin et al., 1995). Conduction velocity in atrial and ventricular cardiomyocytes was 0.3 to 0.4 m/s (Shih, 1994). As we utilized optical recording as a substitute for microelectrode measurement in order to acquire electrophysiological parameters in this study, absolute values including RMP and dV/dt_max_ were not available. However, it has been reported that the RMP in atrial-like hESC-CMs treated with ATRA was -56 ± 2 mV (Laksman et al., 2017), while the RMP in isolated adult human atrial cardiomyocytes was shown to be -74 ± 6 mV (Dawodu et al., 1996). Therefore, it remains challenging to utilize an atrial-like hiPSC-CM preparation to develop a model for atrial arrhythmia and drug discovery.

Fourthly, the nodal-specific genes *SHOX2* and *HCN4* were significantly upregulated in ATRA-treated hiPSC-CMs compared with those in control hiPSC-CMs (Supplementary Figure 3), in addition to the significantly higher expression of atrial specific genes (Figure 4). In particular, ATRA-treated hiPSC-CMs displayed significantly shorter CL than those of control hiPSC-CMs (Figure 5B), suggesting that CMs with nodal-like phenotype were partially included in ATRA-treated hiPSC-CMs. *NKX2.5* and *SHOX2* were shown to have a mutually antagonistic effect in mice, with the former activating the commitment to working myocardium and the latter promoting a pacemaker-like program (Ye et al., 2015). *NKX2.5*, a main transcriptional factor of working myocardium, upregulates the expression of downstream genes including *SCN5A* but represses the expression of *HCN4* (Barbuti and Robinson, 2015; Vedantham, 2015). In the present study, ATRA-treated hiPSC-CMs showed significantly lower expression of *SCN5A*, with a tendency toward a slightly lower value of *NKX2.5* in ATRA-treated hiPSC-CMs (although the difference was not significant, *P* = 0.127, Supplementary Figure 3), also suggestive of a certain proportion of a nodal-like fraction in the ATRA group. The availability of endogenous RA during early development in vertebrates depends mainly on the local distribution of retinaldehyde dehydrogenases (RALDHs), which comprise major synthesizing enzymes of endogenous RA. RALDHs are predominantly expressed in trunk somite mesoderm during early mouse embryonic stages, with the activity of RA synthesized by RALDHs consequently establishing a gradient declining anteriorly owing to the diffusivity of RA (Cunningham and Duester, 2015). In turn, this anterior-decreasing RA-gradient affects the regional characterization of the embryonic heart. The posterior region of the embryonic heart, which is subject to substantial influence from RA, eventually corresponds approximately to the region in which not only primitive atrium but also sinus venosus including the sinus node are located, as cardiac development progresses. Accordingly, it is likely that RA impels hiPSC-CMs into nodal-like as well as atrial-like properties, comparable to posteriorization during *in vivo* cardiac development. Therefore, it would be desirable to develop a more rigorous induction method focusing on hiPSC-CMs with an atrial-specific phenotype free from a nodal-like fraction in the future. In addition, distinct criteria for cardiac phenotyping remain to be well established. In the present study, MLC2a and MLC2v were employed in immunofluorescence staining for atrial and ventricular identification. MLC2a, the atrial isoform of MLC2, is ubiquitously expressed in all cardiac chambers during the fetal development stage, whereas its expression is localized in the atrium at the adult stage (de Sousa Lopes et al., 2006). In comparison, MLC2v, the ventricular isoform of MLC2, exhibits ventricular-specific expression perpetually from the fetal stage to adulthood, except for at very early stages of cardiogenesis (de Sousa Lopes et al., 2006). Therefore, the expression of MLC2a in hiPSC-CMs is upregulated during early cardiac differentiation stages independent of cardiac phenotype, whereas cells positive for MLC2a are restricted to atrial-like hiPSC-CMs with progression toward differentiation. In contrast, few ventricular-like hiPSC-CMs exhibit positive staining for MLC2v during the immature differentiation phase, although the expression of MLC2v is increased in ventricular-like hiPSC-CMs with maturation. In the present study, MLC2a and MLC2v displayed moderate expression in control hiPSC-CMs (Figure 3A,C), suggesting that some hiPSC-CMs with ventricular-like properties remained negative for MLC2v owing to the immaturity. In turn, NR2F1 (Nuclear Receptor subfamily 2, group F, member 1) and NR2F2 (Nuclear Receptor subfamily 2, group F, member 2), also known as COUP-TF1 (Chick Ovalbumin Upstream Promoter-Transcription Factor 1) and COUP-TF2 (Chick Ovalbumin Upstream Promoter-Transcription Factor 2), respectively, comprise transcriptional factors upregulated by RA signaling during atrial commitment. RA facilitates atrial-specific gene expression such as *KCNA5* and *KCNJ3* through the activation of NR2Fs (NR2F1/2) (Devalla et al., 2015). The expression of *NR2F1* was significantly upregulated in ATRA-treated hiPSC-CMs relative to that in control hiPSC-CMs in the present study (Figure 4), suggesting that immunofluorescence staining with NR2F1/2 might serve as a more distinct marker for atrial specification compared with MLC2a, as shown by Devalla et al. (Devalla et al., 2015). Moreover, we substituted optical recordings for microelectrode measurements in order to estimate electrical phenotype of hiPSC-CMs, and OAP recordings disclosed that ATRA-treated hiPSC-CMs in the current study exhibited atrial-like properties represented by shorter APD. However, as we performed optical recordings at a relatively low sampling rate in the present study, so it was likely to be rather insufficient for the precise acquisition of d(-F)/dt_max_.

Fifthly, in the present study we did not elucidate the mechanism by which human atrial Fbs deteriorated the stability of electrical conduction, because we placed emphasis on observation of the effects of Fbs on electrical propagation. Fbs express connexins to establish electrical heterocellular coupling with the adjacent CMs in the mammalian heart (Goldsmith et al., 2004; Kohl and Camelliti, 2012), functioning as an electrotonic and capacitive loading as well as providing a simple physical barrier against electrical conduction (Kucera et al., 2017). Conduction velocity on a neonatal rat CM monolayer significantly decreased when electrotonically loaded with cardiac Fbs (McSpadden et al., 2009). Additionally, Fbs are transformed into myofibroblasts, also termed activated fibroblasts, in response to pathological conditions such as mechanical overload or inflammation. Myofibroblasts have a larger cell membrane capacitance (Chilton et al., 2005) and exhibit more abundant expression of connexins compared with Fbs (Miragoli et al., 2006), contributing to the substantial arrhythmogenesis including conduction disturbance that occurs upon augmenting the electrotonic and capacitive loading effect on CMs (Rohr, 2012; Kucera et al., 2017). In the adult mammalian hearts, non-cardiomyocytes are larger in number than cardiomyocytes (Baudino et al., 2006), and cardiac fibroblasts constitute a major component of non-cardiomyocytes in the human heart (Bergmann et al., 2015), thereby fibroblasts with small membrane capacitance are thought to have effects on electrical conduction. However, in our study, the ratio of the number of Fbs to the number of CMs plated was relatively low compared with the ratio in the human heart, so it was uncertain to what extent the Fbs in our model impacted on electrical propagation. Paracrine effects of cardiac Fbs can also deteriorate electrical conduction. Cardiac Fb-conditioned medium has been shown to have an influence on electrophysiological properties in neonatal rat ventricular cardiomyocytes, such as the slowing of conduction velocity, less negative RMP, and APD prolongation mainly through the downregulation of *SCN5A, KCNJ2*, and *KCDN3*, respectively (Pedrotty et al., 2009). LaFramboise WA et al. reported that various paracrine factors, including vascular endothelial growth factor (VEGF), interleukin (IL)-6, tumor necrosis factor (TNF)-α, and transforming growth factor (TGF)-β, etc., were elevated in cardiac Fb-conditioned medium (LaFramboise et al., 2007). Actually, it has been shown that neonatal rat atrial cardiomyocytes exposed to TGF-β1 displayed a significant reduction in voltage-dependent Na^+^ current and delayed rectifier K^+^ (Ramos-Mondragon et al., 2011). Therefore, cardiac fibroblasts in our setting potentially exerted effects on electrical propagation also by paracrine mechanism. Moreover, pathological condition such as cardiac overload promotes collagen synthesis and the distribution pattern of fibrosis in PVs and atrium can provide AF substrate via the anatomical obstacle. The clustering of Fbs in culture are more likely to recapitulate *in vivo* collagen deposition and fibrosis, although Fbs were randomly plated in our setting. Conduction disturbance is more conspicuous when fibrous tissue is located between cardiomyocytes in perpendicular to the longitudinal direction (Yue et al., 2011). Thus, the clustering of Fbs in such a pattern in coculture with atrial-like hiPSC-CMs is more likely to provoke impaired conduction on an *in vitro* atrial arrhythmia model. The fibrosis pattern exhibits a highly individual variation, so we had difficulty in constructing an *in vitro* human physiological or pathological relevant model exactly incorporating this factor. If a new technique (for example, 3D printing) enabling the incorporation of fibrosis pattern into an *in vitro* atrial arrhythmia model is available in the future, the recapitulation of microreentry on atrial-like hiPSC-CM monolayers might be without serious difficulty. Thus, further studies are needed to clarify the roles of atrial Fbs and fibrosis in electrical propagation on atrial-like hiPSC-CM preparation, in order to construct a more elaborate human atrial arrhythmia model in the future.

Finally, the significant differences in Tables 1, 2 using Fisher’s exact test were identified only when the percentile was 0 vs. 100. This low statistical power shall be attributed to the small sample size, and this could be a potential limitation in the present study. Moreover, in this study we conducted all procedures for OAP measuring and optical mapping experiment using a voltage-sensitive dye, FluoVolt^TM^ with the modified filter setting (the detailed procedure is described in Section “Optical Membrane Potential Imaging”). Although the standard FITC filter setting is recommended for FluoVolt signal acquisition by the manufacturer, in our experimental system, however, the standard setting did not yield acceptable signal-to-noise ratio. On the other hand, our modified filter setting, which is originally used for the detection of voltage-sensitive dye signal with long-wavelength region such as Di-8-ANEPPS, displayed increased stability of signal-to-noise ratio during repeated optical recordings. To our knowledge, this is the first report to introduce the alternative filter setting for FluoVolt^TM^ with a sufficient visualization of the electrical propagation patterns, which was the principal objective in the present study, although the mechanism for the unexpectedly unique characteristic of the dye remains to be elucidated.

In summary, geometrical patterning with an abrupt narrow-to-wide transition under high frequency stimuli preferentially provokes impaired electrical conduction in an *in vitro* 2D atrial-like hiPSC-CM monolayer. Constituent cell heterogeneity including non-CMs represented by atrial Fbs further deteriorated the integrity of electrical conduction relative to that of an atrial-like hiPSC-CM alone preparation. hiPSC-CMs with atrial-like phenotype represent a potent tool as a human-based atrial CMs source, with our findings further suggesting that the present model incorporating a portion of the structural and constituent features within PVs and the PVs-LA transition might evolve into an *in vitro* platform for pathophysiology and drug screening of human AF in the future. Further studies are required to improve the quality of atrial-like hiPSC-CMs and develop an optimal model reflecting the proarrhythmic substrate of AF.

## Materials and Methods

All experiments were approved by the Osaka University Institutional Review Board and performed under the guidelines of the Osaka University Committee (Authorization No. 04033).

### Cell Culture, Directed Cardiac Differentiation, and Metabolic Purification

We utilized 201B7 hiPSCs (RIKEN BRC Cell Bank, Tsukuba, Japan) as a pluripotent cell source from a healthy human. The hiPSCs were maintained under a feeder/serum/xeno-free condition as previously reported (Nakagawa et al., 2014). Briefly, the undifferentiated cells were cultured on 0.5 μg/cm^2^ iMatrix-511^TM^ (Nippi, 892021) in StemFit^®^ AK02N medium (Ajinomoto, AK02N) and passaged enzymatically at 80 to 90% confluence.

For the induction of cardiac differentiation, we modified the original monolayer-based directed cardiac protocol with stage-specific activation and inhibition of Wnt/β-catenin signaling during differentiation (Lian et al., 2013); the protocol used is shown in Figure 1. Briefly, hiPSCs at 80 to 90% confluence were dissociated enzymatically into single cells using Accutase (Innovative cell technology, AT104) and plated on iMatrix-511^TM^-coated 24 well plates in StemFit^®^ AK02N medium containing 10 μM Y-27632 (Wako, 036-24023), a Rho-associated protein kinase inhibitor, at day -4. The next day, the hiPSC medium was replaced with AK02N medium without Y-27632, whereupon the hiPSCs expanded up to 70 to 90% confluence over the following 3 days. At day 0, RPMI 1640 Medium (Thermo Fisher Scientific, 11875-093) containing B-27 supplement minus insulin (Thermo Fisher Scientific, A1895601) and 9 μM CHIR-99021 (Selleckchem, S2924), a chemical compound of Wnt signaling activation, was used to differentiate the hiPSCs into mesoderm cells. Matrigel (Growth Factor Reduced, Corning, 354230), an extracellular matrix product, was added at days -2 and 0 to promote epithelial-mesenchymal transition for efficient cardiac differentiation (Zhang et al., 2012). For cardiac mesoderm specification, 5 μM IWP2 (Tocris, 3533), a chemical compound of Wnt signaling inhibition, was applied at day 3. To elicit atrial specific commitment, the culture system was treated with 1 μM ATRA (Sigma Aldrich, R2625) during days 5 to 9. From day 7, we used RPMI 1640 Medium containing B-27 supplement (Thermo Fisher Scientific, 17504-044) as basal medium, except for metabolic selection period as mentioned below. Spontaneous beating was initiated almost by day 14. Purification of CMs was conducted by metabolic selection with a glucose and glutamine-free medium containing 0.1% bovine serum albumin (Wako, 037-23372), 5 mM lactic acid (Wako, 128-00056), and 0.73 mM L-ascorbic acid 2-phosphate (Sigma Aldrich, A8960-5G) for 3 days per course for two courses (Burridge et al., 2014; Tohyama et al., 2016). The CM preparations were maintained from day 23 to 49, at which point they were isolated for downstream experiments such as immunofluorescence staining, RNA analysis, and electrophysiological study.

In addition, we purchased commercially available human atrial Fbs isolated from the atrium of a normal adult (Lonza, CC-2903), and cultured them according to the supplier’s protocol. The Fbs were employed in the coculture system with atrial-like hiPSC-CMs as described in Section “Optical Mapping.”

### Immunofluorescence Staining

Cultured cells were fixed with 4% paraformaldehyde in phosphate buffered saline (PBS) (-) for 15 min at 4°C, permeabilized with 0.1% Triton-X in PBS(-) for 15 min at room temperature (RT), and then blocked with 10% donkey serum and 0.1% Triton-X in PBS(-) for 60 min at RT. Primary antibodies were reacted overnight at 4°C. The dilutions of primary antibodies were as follows: anti-Oct3/4 (1:200, R&D, AF1759), anti-Nanog (1:200, R&D, AF1997), anti-SSEA-4 (1:200, R&D, MAB1435), anti-Brachyury (1:100, R&D, AF2085), anti-cardiac troponin T (1:200, Lab Vision, MS-295-P1), anti-MLC2a (1:200, Synaptic Systems, 311011), anti-MLC2v (1:200, ProteinTech Group, 10906-1-AP), anti-P4HB (1:250, Abcam, ab137110), and anti-vimentin (1:200, Abcam, ab11256). Secondary antibodies were applied for 60 min at RT in the following dilution: donkey anti-mouse IgG (H+L) Alexa Fluor^®^ 488, donkey anti-mouse IgG (H+L) Alexa Fluor^®^ 594, donkey anti-rabbit IgG (H+L) Alexa Fluor^®^ 488, donkey anti-rabbit IgG (H+L) Alexa Fluor^®^ 594, donkey anti-goat IgG (H+L) Alexa Fluor^®^ 488, donkey anti-goat IgG (H+L) Alexa Fluor^®^ 568 (all 1:500, Thermo Fisher Scientific). Cell nuclei were stained with TOPRO-3 (Thermo Fisher Scientific, T3605) or Hoechst 33342 (Dojindo, H342). Fluorescence images were obtained using an LSM700 confocal microscope (Zeiss, Tokyo, Japan) or Operetta^®^ high content imaging system (PerkinElmer, Yokohama, Japan) and analyzed using Harmony^®^ analysis software (PerkinElmer, Yokohama, Japan).

### Quantitative Reverse Transcription Polymerase Chain Reaction (qRT-PCR)

Total RNA was extracted using the RNeasy^®^ Plus Mini Kit (Qiagen, 74136) and quantified with a NanoDrop 2000 Spectrophotometer (Thermo Fisher Scientific). cDNA was synthesized using the SuperScript^TM^ VILO^TM^ cDNA Synthesis Kit (Thermo Fisher Scientific, 11754-250). qRT-PCR was performed by using TaqMan^TM^ Fast Advanced Master Mix (Thermo Fisher Scientific, 4444557) on a ViiA^TM^7 Real-time PCR system (Thermo Fisher Scientific). The genes were analyzed using the following TaqMan^®^ gene expression assays (Thermo Fisher Scientific): *MYL7* (Hs01085598_g1), *MYL2* (Hs00166405_m1), *TNNT2* (Hs00943911_m1), *NPPA* (Hs00383230_g1), *IRX4* (Hs00212560_m1), *SLN* (Hs01888464_s1), *PITX2* (Hs04234069_mH), *NR2F1* (Hs00818842_m1), *TBX5* (Hs00361155_m1), *KCNA5* (Hs00969279_s1), *KCNJ3* (Hs04334861_s1), *CACNA1C* (Hs00167681_m1), *GJA5* (Hs00270952_s1), *GJA1* (Hs00748445_s1), *NKX2.5* (Hs00231763_m1), *SCN5A* (Hs00165693_m1), *KCNJ2* (Hs01876357_s1), *SHOX2* (Hs00243203_m1), *HCN4* (Hs00175760_m1), and 18S ribosomal RNA (Hs99999901_s1). Gene expression values were defined as relative to ATRA-untreated hiPSC-CMs (control hiPSC-CMs) using the delta-delta Ct method, normalizing with 18S ribosomal RNA, which is a housekeeping gene, as an internal control. All qRT-PCR experiments included two technical replicates per each independent sample.

### Optical Membrane Potential Imaging

For optical membrane potential imaging, preparations of hiPSC-CMs were loaded for 30 min at RT with a voltage-sensitive dye using the FluoVolt^TM^ Membrane Potential Kit (Thermo Fisher Scientific, F10488), the excitation and emission wavelength of which are 522 and 535 nm, respectively. Then, 10 μM blebbistatin (Wako, 027-17043), an excitation-contraction uncoupler, was applied to avert motion artifact. For FluoVolt signal acquisition, the loaded sample was excited with a 530 nm light emitting diode (LED), and a filter setting (excitation, 520/35–25 nm; emission, long-passed at 580 nm) was used. All experiments were performed at 37°C under aerial condition. OAP imaging was acquired at a sampling rate of 5 or 10 ms per frame using the MiCAM02 imaging system (Brainvision, Tokyo, Japan) equipped with a high-speed CMOS camera, the field-of view and spatial resolution of which were 5.76 × 4.8 mm and 30 × 30 μm, respectively. OAP parameters including average CL, d(-F)/dt_max_, and APD were calculated using OriginPro 8.6J software (LightStone, Tokyo, Japan). Peak to peak interval was used to calculate beating rate. d(-F)/dt_max_, a surrogate marker of the dV/dt_max_ recorded using patch clamp technique, was defined as the maximal instantaneous change in fluorescence normalized to the difference in fluorescence between the baseline in diastolic phase and maximal amplitude of the upstroke, as previously reported (Leyton-Mange et al., 2014). APD_x_ (*x* = 20, 50, 90) was measured as the interval from the timing of 50% maximal upstroke amplitude to the time point of x% of repolarization, as previously described (Shinnawi et al., 2015). As APD can be affected by contraction rate, we used Bazett’s formula [=APD/√(average cycle length/1000)] to transform raw APD into cAPD. All parameters were computed as an average of five consecutive waveforms at each region of interest (ROI), and OAP parameters from independent samples were designated as an average of several ROIs selected in the sample.

### Geometrical Patterning Culture Model

To construct a culture model with geometrical narrow-to-wide patterning, we utilized a commercially available silicone rubber sheet (MISUMI, RBAMF6H5) that contains two separate circular holes with a radius of 6 mm. The silicone was manually engraved at the area between the two circular holes to create a linear strand configuration connecting the holes (Figure 6A). The width of a strand was defined as 1 to 2 mm for the geometrical patterning model with narrow isthmus or 12 mm for the non-isthmus model. As a result, the 1 to 2 mm strand pattern exhibited an abrupt narrow-to-wide transition structure at the border between the strand and the right circular area, whereas the 12 mm strand model was without such a geometrical change. The left circular area in the narrow isthmus model or the left area in the non-isthmus model was utilized as a site for electrical field stimulation. The fabricated silicone mold was attached on a 100 mm culture dish (Corning, 353003) for optical mapping experiments as described in Section “Optical Mapping.”

### Optical Mapping

For optical mapping, the fabricated plate was precoated with Geltrex^®^ (Thermo Fisher Scientific, A1413302), and then a mixture of atrial-like hiPSC-CMs and human atrial Fbs was transferred at a total density of 6.0 × 10^5^ cells per cm^2^ on the plate in ratios of 100, 70, or 0% hiPSC-CMs to 0, 30, or 100% Fbs. The coculture system was cultivated for approximately 7 to 10 days, at which point robust synchronized contraction was observed except for in the 100% Fb group, after which optical mapping was performed as follows. The cell preparation was loaded with FluoVolt as described in Section “Optical Membrane Potential Imaging.” A bipolar electrode was inserted into the pacing site to generate electrical excitation, intended to propagate through a strand into the area ahead. Electrical field stimulation was delivered using MyoPacer EP (IonOptix, Westwood, MA, United States), beginning at a slightly higher frequency than that of the spontaneous beating, then increased by 0.5 Hz up to the frequency that exhibited local capture failure in the strand area. The pacing condition was at 20 V with a pulse width of 10 ms; no local capture failure was observed in the setting except for the preparation only sample, consisting of 100% Fbs. The recording site of optical mapping, illustrated by the hollow red square in Figure 6A, was within the region mainly from the abrupt narrow-to-wide transition to the slightly distal area in the narrow isthmus model or the almost corresponding area based on coordinates in the non-isthmus model. Optical mapping was implemented using MiCAM02 (Brainvision, Tokyo, Japan) under the same condition as for optical membrane potential imaging. The acquisition data were analyzed using BV_Ana software (Brainvision, Tokyo, Japan).

For the assessment of the geometrical effect on electrical conduction, we defined the maintained or impaired conduction preparation as follows: the former was sustaining 1 to 1 conduction from a strand to the further distal area at each pacing frequency, whereas the latter was exhibiting conduction disturbance from a strand into the distal area at the pacing rate equal to or less than the maximum rate capable of local capture in the strand. The preparations that reached the local capture failure in a strand region at a delivered pacing frequency and those that had already exhibited impaired conduction from a strand into the distal region at the lower pacing frequency were also included in the impaired conduction group based on delivered pacing frequency. Success rate of electrical conduction into the distal region at each pacing frequency was determined as the percentage of maintained conduction preparations at the pacing rate.

### Processing of Optical Mapping Data

The processing and analysis of the acquired optical data were conducted using BV_Ana software (Brainvision, Tokyo, Japan), referring to a previous report (Laughner et al., 2012). In brief, the optical signal from an object loaded with the voltage-sensitive dye was normalized to isolate it from background pixels less than the threshold, which was defined as 10% of the highest signal intensity within the field. The normalized signal, ΔF/F, underwent spatial filtering of 3 × 3 uniform binning followed by inversion and fast Fourier transform with a cutoff value at one tenth of the sampling rate frequency, to omit noise signal with a high frequency component. Polynomial fitting was employed for drift correction. To develop the activation map from the acquired optical membrane potential, local activation time was defined as the time at which OAP achieved the peak intensity at each point.

### Statistical Analysis

All data are expressed as the means ± standard deviation (SD). Sample sizes represent the number of independent biological replicates from different sample preparations. The continuous variable between two independent groups or more than two groups was compared by using the unpaired *t*-test or one-way ANOVA followed by Tukey’s *post hoc* test, respectively. The comparison of a binary variable between two independent groups was estimated with Fisher’s exact test. *P*-values of <0.05 were considered statistically significant. N.S. indicates not significant. All statistical analyses were conducted with EZR (Saitama Medical Centre, Jichi Medical University, Saitama, Japan) (Kanda, 2013), which is a graphical user interface for R (The R Foundation for Statistical Computing, Vienna, Austria).

## Ethics Statement

All experiments were approved by the Osaka University Institutional Review Board and performed under the guidelines of the Osaka University Committee (Authorization No. 04033).

## Author Contributions

HN and J-KL conceptualized the study, designed and conducted the experiments, analyzed the data, and wrote the manuscript. KeM, KiM, HY, JL, SaT, YH, JD, ShT, KH, SM, YoS, IK, and YaS analyzed the data and reviewed the manuscript. IK and YaS supervised the study. All authors approved the final version of the manuscript.

## Conflict of Interest Statement

J-KL received a Joint Research Grant (Alpha MED Scientific, Inc., SCREEN Holdings Co., Ltd., Sumitomo Dainippon Pharma Co., Ltd.). YH, JD, and ST were employed by Sumitomo Dainippon Pharma Co., Ltd. The remaining authors declare that the research was conducted in the absence of any commercial or financial relationships that could be construed as a potential conflict of interest.

## Abbreviations

ATRA: all-*trans* retinoic acid
A.U.: arbitrary unit
OAP: optical action potential
PLA: posterior left atrium
RLU: relative luminescent units
SPB: septopulmonary bundle
