# Supplementary figures and images for: Geometrical Patterning and Constituent Cell Heterogeneity Facilitate Electrical Conduction Disturbances in a Human Induced Pluripotent Stem Cell-Based Platform: An In vitro Disease Model of Atrial Arrhythmias
Source: Front Physiol. 2019 Jun 27;10:818. doi: 10.3389/fphys.2019.00818 (PMC6610482; doi:10.3389/fphys.2019.00818)

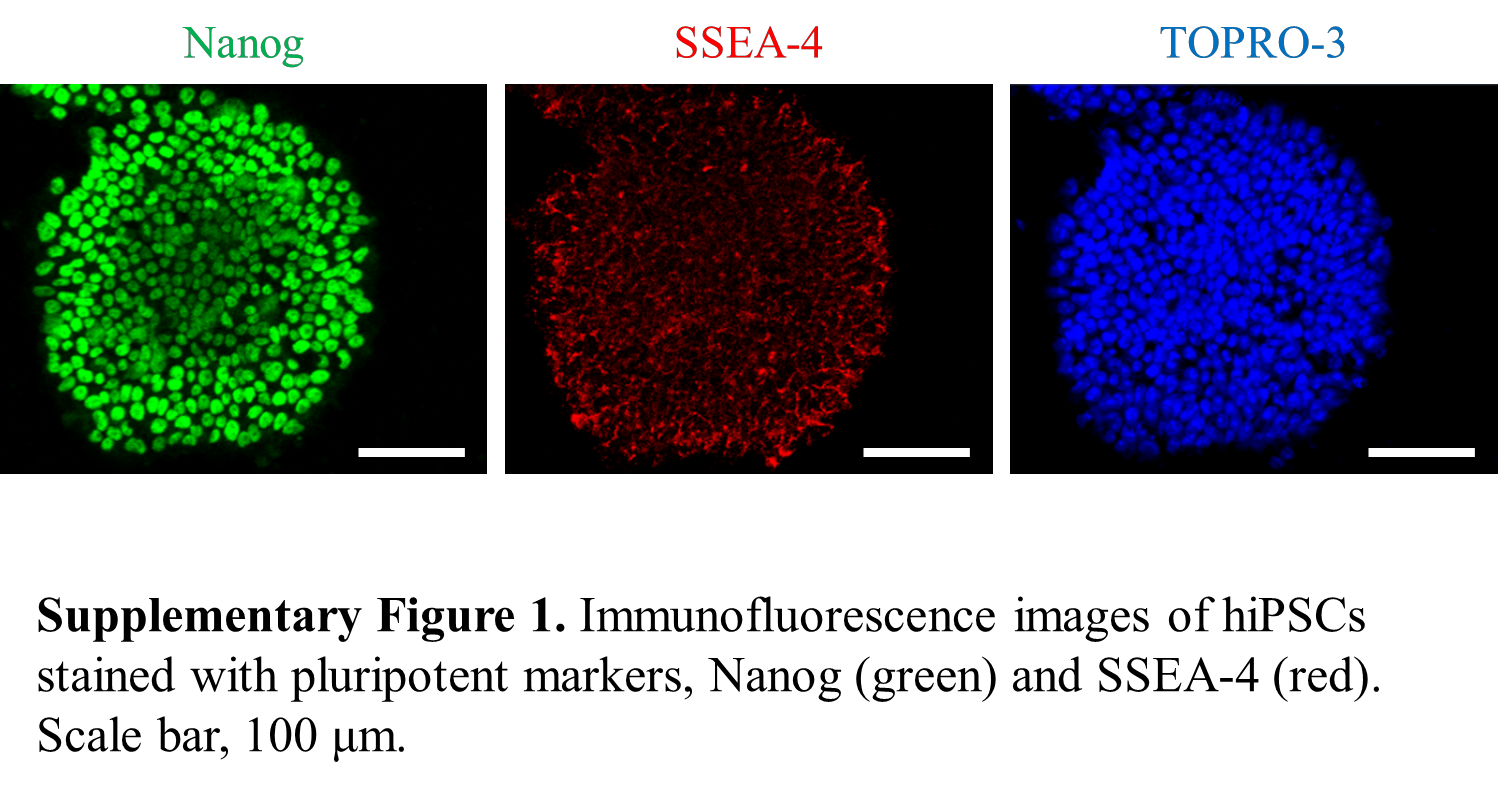

Supplement: Supplementary file 12 [file Image_1.TIF]

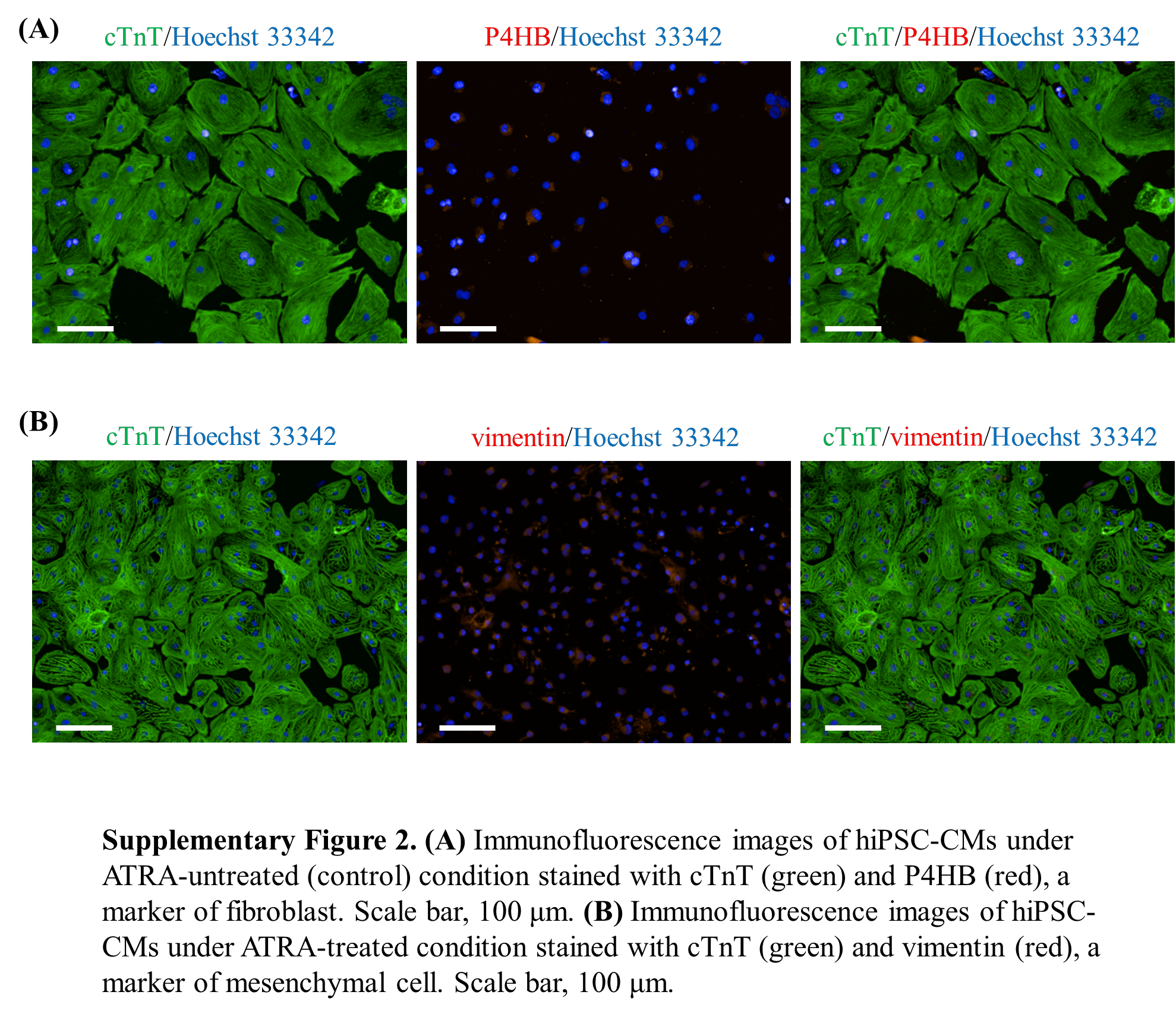

Supplement: Supplementary file 13 [file Image_2.TIF]

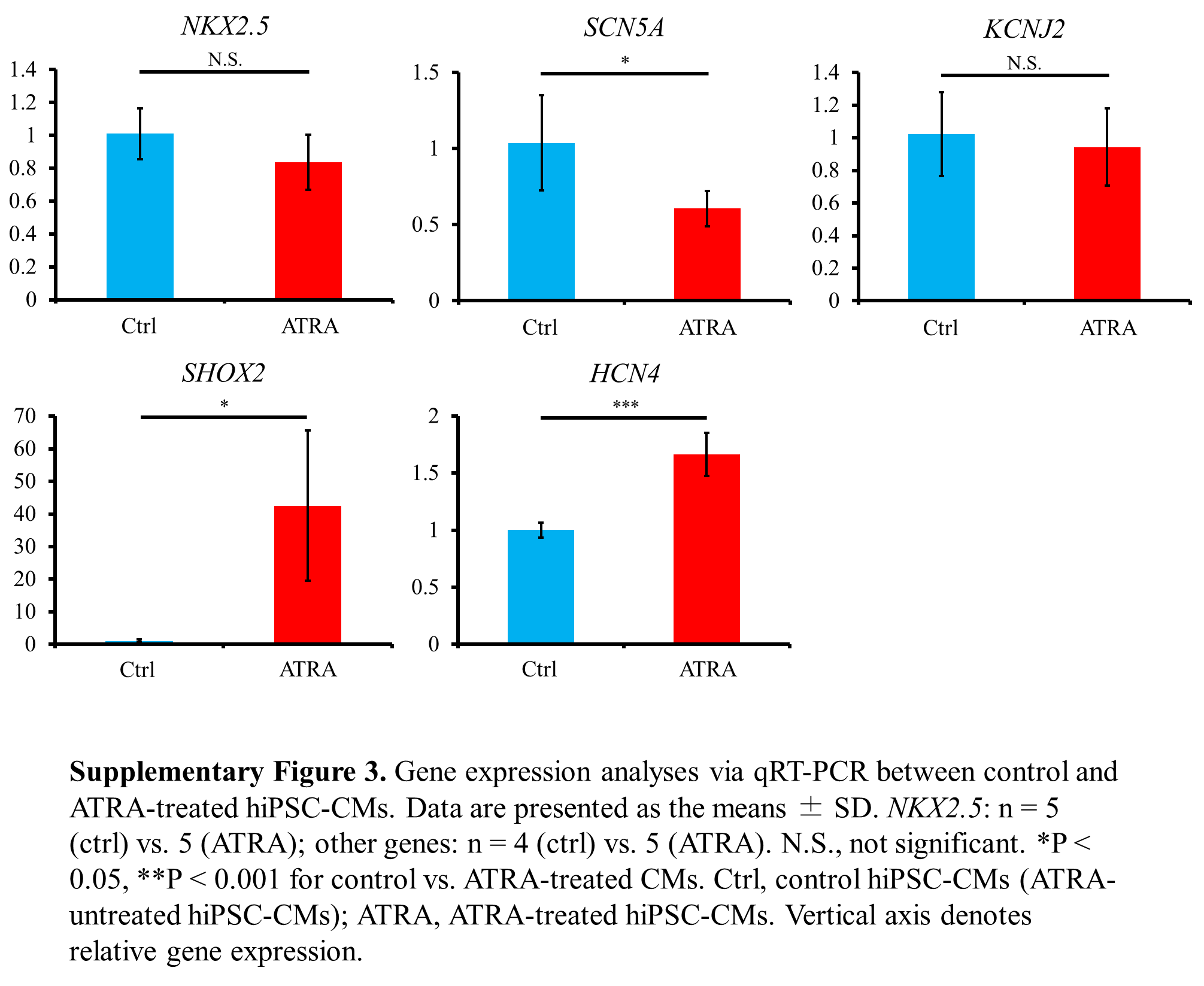

Supplement: Supplementary file 14 [file Image_3.TIF]

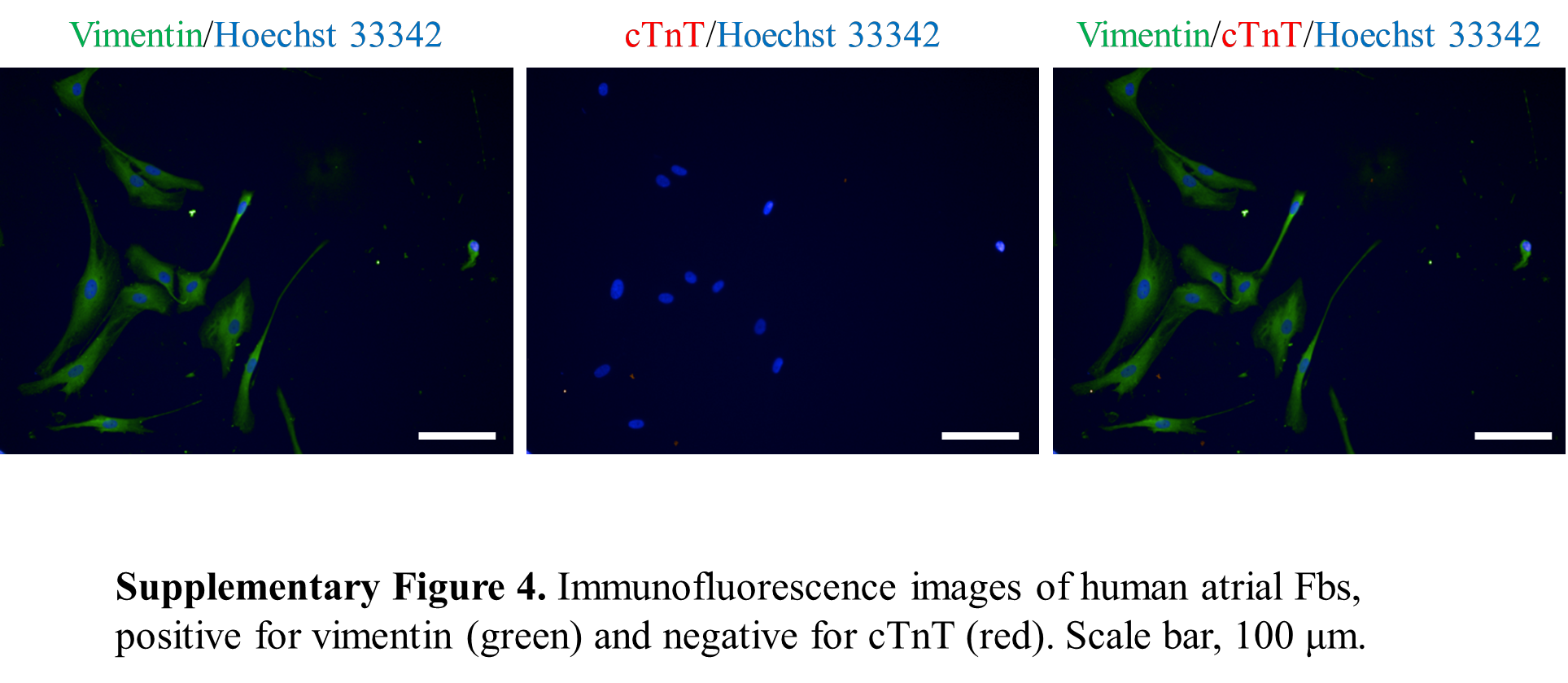

Supplement: Supplementary file 15 [file Image_4.TIF]

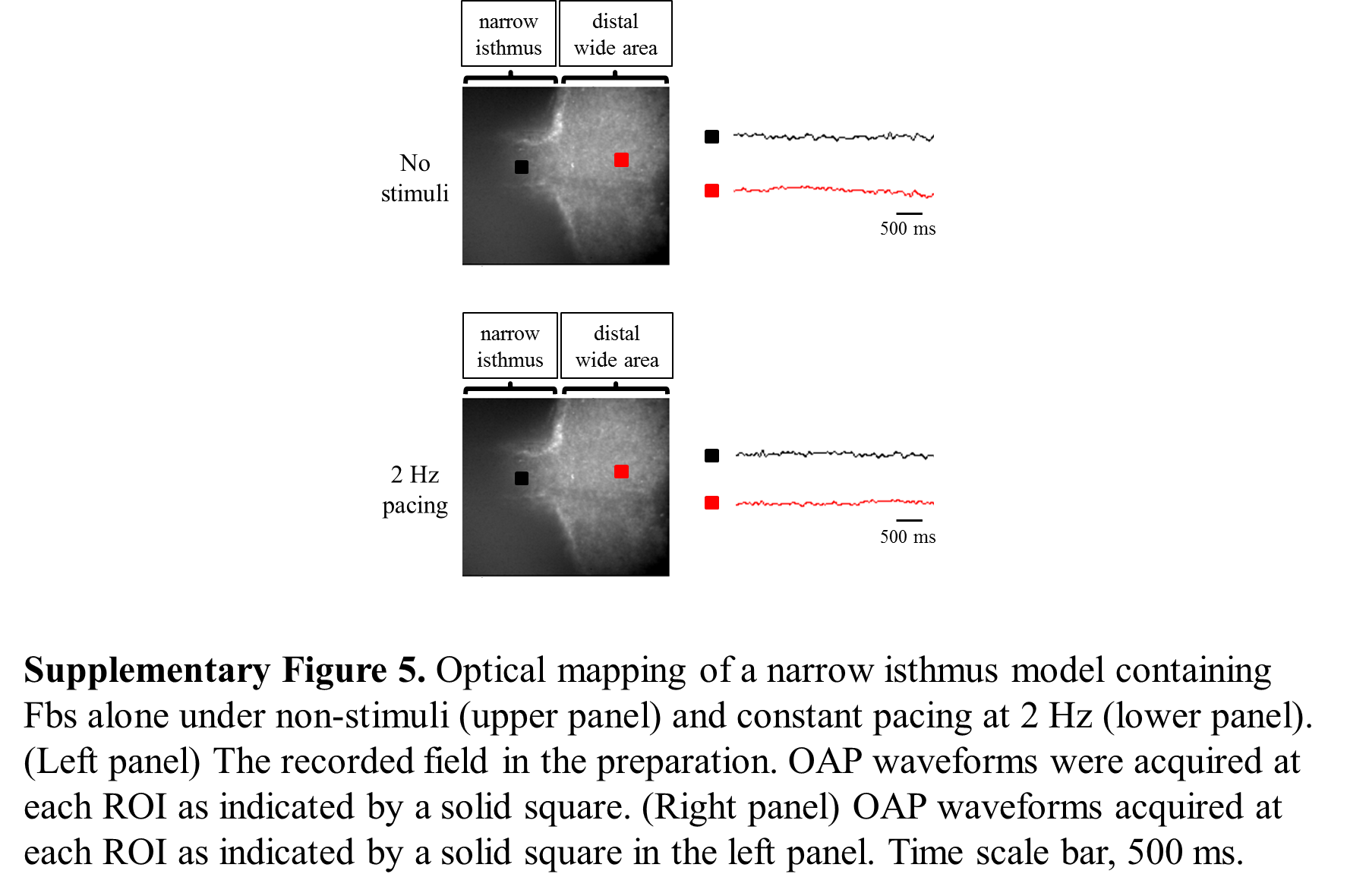

Supplement: Supplementary file 16 [file Image_5.TIF]

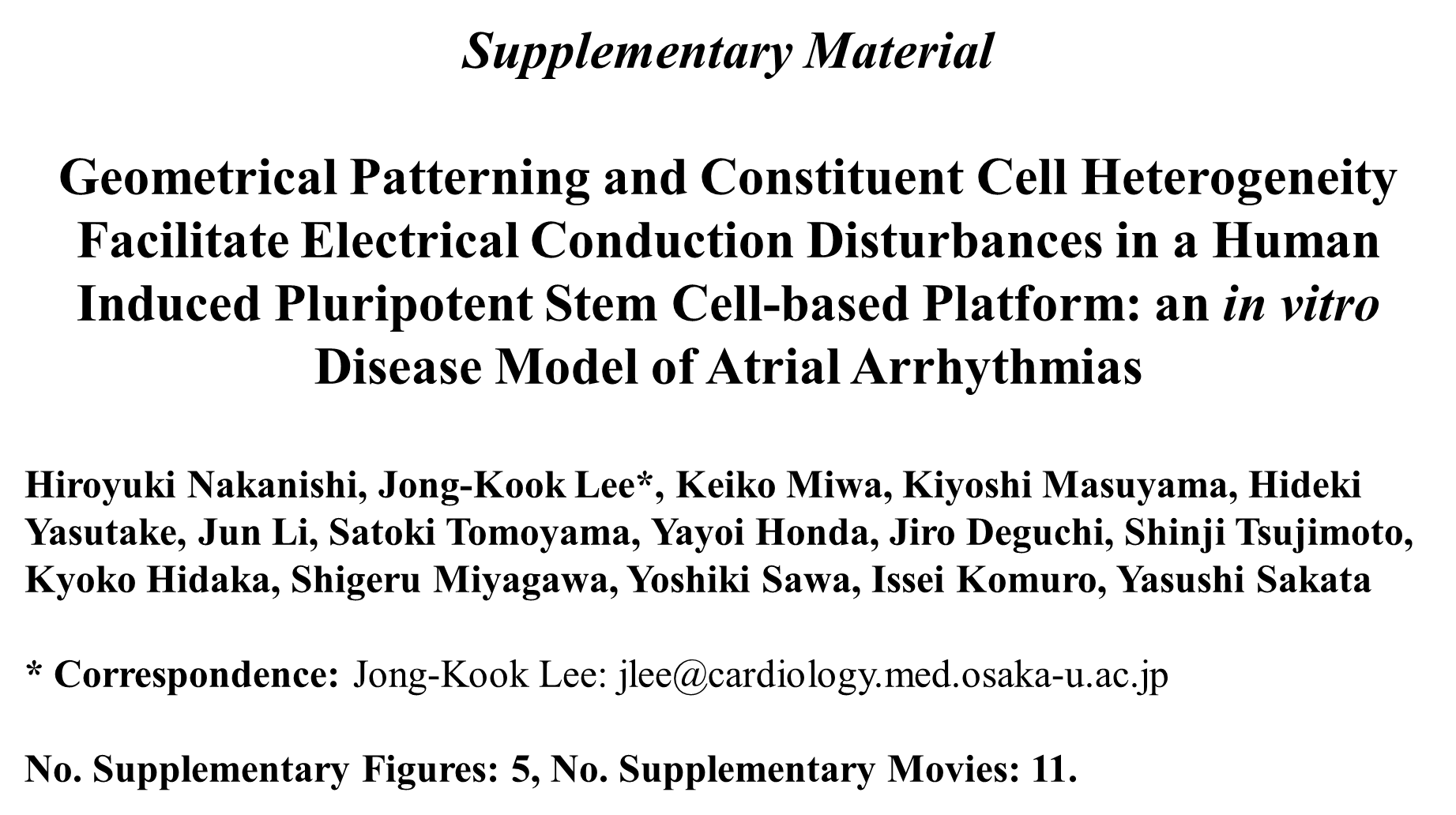

Supplement: Supplementary file 17 [file Image_6.TIF]

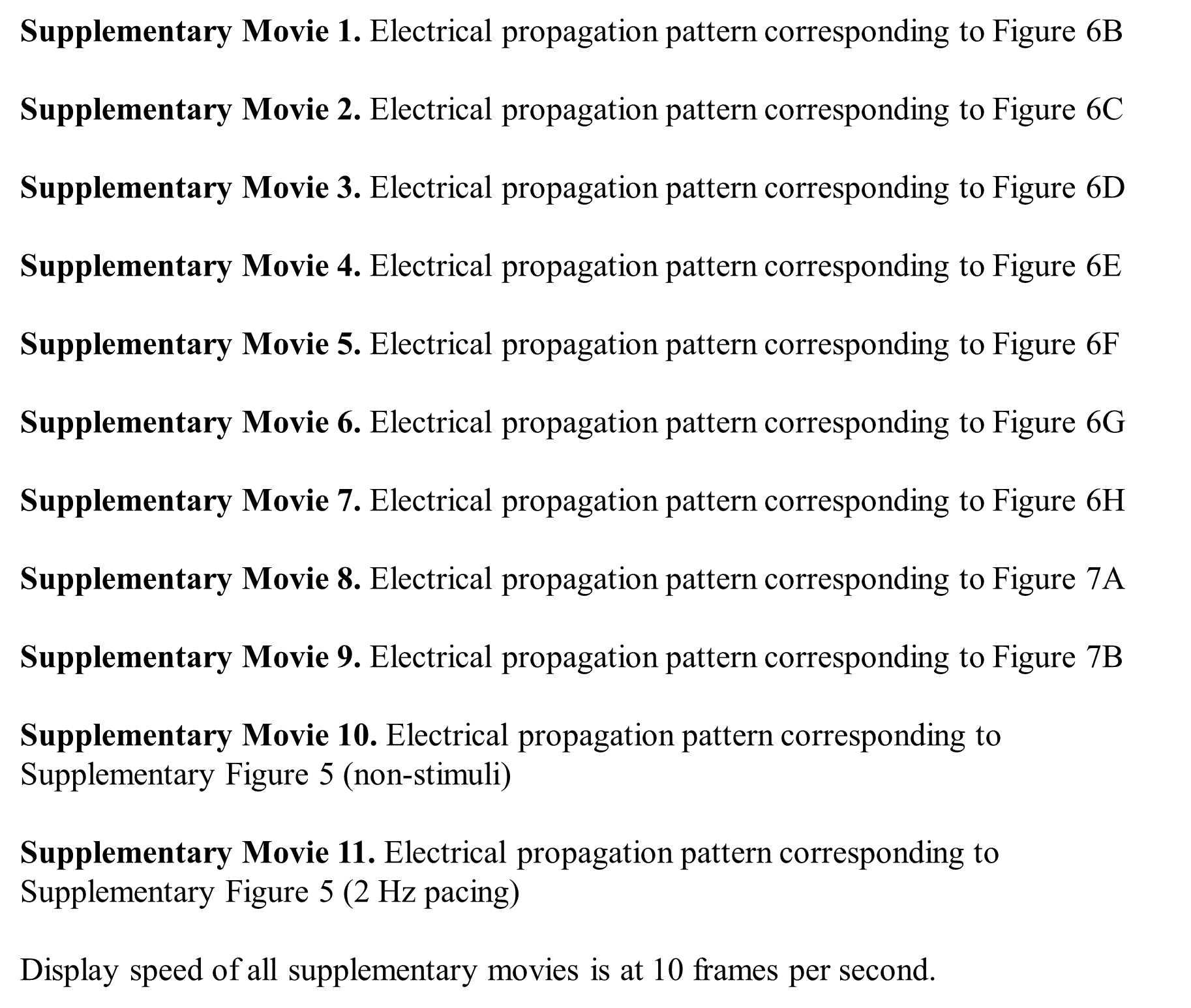

Supplement: Supplementary file 18 [file Image_7.TIF]
